# Oil-Water Biphasic Metal-Organic Supramolecular Gel for Lost Circulation Control: Formulation Optimization, Gelation Mechanism, and Plugging Performance

**DOI:** 10.3390/gels12010074

**Published:** 2026-01-15

**Authors:** Qingwang Li, Songlei Li, Ye Zhang, Chaogang Chen, Xiaochuan Wu, Menglai Li, Shubiao Pan, Junfei Peng

**Affiliations:** 1 National Joint Engineering Research Center for Shale Gas Exploration and Development, Chongqing Institute of Geology and Mineral Resources, Chongqing 400042, China; amazingwxl@163.com (Q.L.); hsiaochuanwu@hotmail.com (X.W.); lml_90@sina.com (M.L.); pan_shubiao@163.com (S.P.); pengjunfeihuihui@163.com (J.P.); 2 Key Laboratory of Shale Gas Exploration, Ministry of Land and Resources, Chongqing Institute of Geology and Mineral Resources, Chongqing 400042, China; 3 China North Chemical Research Academy Group Co., Ltd., Beijing 100080, China; 4 Downhole Services Company, Bohai Drilling Engineering Company Limited (BHDC), Renqiu 062552, China; lisonglei126@126.com; 5 Sichuan Energy Investment Oil and Gas Exploration and Development Co., Ltd., Chengdu 610000, China; chenchaogang2008@126.com

**Keywords:** metal–organic supramolecular gel, oil–water biphasic system, self-healing material, lost circulation control, drilling fluid, wellbore plugging

## Abstract

Lost circulation in oil-based drilling fluids (OBDFs) remains difficult to mitigate because particulate lost circulation materials depend on bridging/packing and gel systems for aqueous media often lack OBDF compatibility and controllable in situ sealing. A dual-precursor oil–water biphasic metal–organic supramolecular gel enables rapid in situ sealing in OBDF loss zones. The optimized formulation uses an oil-phase to aqueous gelling-solution volume ratio of 10:3, with 2.0 wt% Span 85, 12.5 wt% TXP-4, and 5.0 wt% NaAlO_2_. Apparent-viscosity measurements and ATR–FTIR analysis were used to evaluate the effects of temperature, time, pH, and shear on MOSG gelation. Furthermore, the structural characteristics and performances of MOSGs were systematically investigated by combining microstructural characterization, thermogravimetric analysis, rheological tests, simulated fracture-plugging experiments, and anti-shear evaluations. The results indicate that elevated temperatures (30–70 °C) and mildly alkaline conditions in the aqueous gelling solution (pH ≈ 8.10–8.30) promote P–O–Al coordination and strengthen hydrogen bonding, thereby facilitating the formation of a three-dimensional network. In contrast, strong shear disrupts the nascent network and delays gelation. The optimized MOSGs rapidly exhibit pronounced viscoelasticity and thermal resistance (~193 °C); under high shear (380 rpm), the viscosity retention exceeds 60% and the viscosity recovery exceeds 70%. In plugging tests, MOSG forms a dense sealing layer, achieving a pressure-bearing gradient of 2.27 MPa/m in simulated permeable formations and markedly improving the fracture pressure-bearing capacity in simulated fractured formations.

## 1. Introduction

Oil-based drilling fluids (OBDFs) possess strong shale inhibition, excellent thermal stability, superior lubricity, and remarkable resistance to contamination, all of which contribute to maintaining wellbore stability [1,2]. They are thus widely used for drilling through complex formations such as mudstone, shale, and salt–gypsum layers. Nevertheless, the use of OBDFs is increasingly constrained in industry because non-aqueous (oil-based/invert-emulsion) fluids and oil-contaminated cuttings are subject to stringent environmental regulation and waste-management requirements, particularly for offshore operations. In many jurisdictions, direct discharge of oil-based drilling fluids is prohibited. In some cases, discharge of associated cuttings is also prohibited. Operators must therefore use total-containment/closed-loop practices and compliant waste handling. Common options include shipping cuttings for onshore treatment, thermal desorption, or cuttings re-injection, rather than routine discharge. Accordingly, OBDF-oriented lost circulation technologies remain practically relevant for scenarios where OBDFs are still necessary or strongly preferred, such as highly reactive shales, salt/gypsum intervals, HPHT wells, and extended-reach drilling.

Compared with water-based drilling fluids, OBDFs exhibit lower flow resistance along formation fractures [3,4,5]. Once lost circulation occurs, sealing becomes much more difficult. This increases non-productive time and operating costs. It also generates larger volumes of oil-contaminated materials. These materials must be contained, managed, and treated to meet regulatory requirements. At present, there is a lack of efficient lost circulation materials and standardized operational guidelines for oil-based drilling fluids. Moreover, conventional plugging materials designed for water-based systems cannot remain stably suspended in oil-based media, leading to a low success rate of field plugging operations [6,7,8]. Under severe loss conditions, cement slurries are often used as plugging agents. However, their performance is easily affected by the oil-based drilling environment [9]. As a result, sealing is often unsatisfactory and may lead to repeated losses or even wellbore collapse in some cases [10,11]. To effectively mitigate lost circulation risks in oil-based drilling, researchers have developed various types of plugging materials, including bridging materials, flexible particulates, in situ crosslinking agents, and intelligent responsive materials [12,13]. However, traditional plugging materials generally exhibit insufficient contact with fracture surfaces. During drilling operations, the plugging layer is prone to damage and detachment under the negative pressure induced by tool movement, leading to the failure of sealing performance. In recent years, supramolecular gels have attracted increasing attention owing to their controllable and reversible structural characteristics [14,15,16], demonstrating great potential for application in lost circulation prevention and plugging in oil-based drilling fluids [17].

Metal–Organic Gels (MOGs) are supramolecular soft materials formed by coordination-capable organic building ligands that self-assemble through noncovalent interactions [18]. Among them, coordination polymer-based MOGs have attracted increasing attention in recent years because of their high designability and multifunctionality [19]. Typically, coordination polymers are formed by metal ions bridged with organic ligands to generate infinitely extended three-dimensional network structures [20]. Within these supramolecular gels, metal–ligand coordination serves as the primary driving force for gel network formation. Interestingly, some MOG systems exhibit thermoreversible behavior opposite to that of conventional organogels—gelation occurs upon heating, while the system remains in a sol state at ambient temperature. Zhang et al. [21] proposed a high-temperature and salt-resistant polymer gel system based on a “metal ion–organic ligand” hybrid crosslinking strategy. This strategy significantly improved the gel’s thermal stability and salt tolerance, expanding its applicability in high-temperature and high-salinity reservoirs. Wang et al. [22] developed a rapidly forming MOG system using phosphate esters and Fe^3+^ ions, which demonstrated excellent thermal resistance and considerable potential for field application. In another study, Wang et al. [23] reported a metal–organic gel with an organic–inorganic dual-network structure and self-healing capability, and discussed its potential in controlling circulation loss during drilling operations. Beyond coordination-driven MOGs, supramolecular gels formed in biphasic systems composed of surfactants, co-surfactants, oil, and water have also been extensively investigated [14,24,25,26]. Under specific compositions, these systems can spontaneously form three-dimensional network structures. Typical examples include bicontinuous or sponge phases. Network formation is driven by noncovalent interactions, such as hydrogen bonding, hydrophobic association, and electrostatic attraction [14,27,28,29]. The resulting supramolecular gels immobilize both oil and water phases within the network, exhibiting high viscosity and reversible rheological behavior. With outstanding structural tunability, stimuli responsiveness, and self-healing capability, supramolecular gels are promising for enhanced oil recovery, lost circulation control, and profile modification and water shutoff in oil and gas field development [30,31,32].

It is worth noting that supramolecular gels constructed via metal–ligand coordination (i.e., metal–organic/coordination-driven gels) have already been explored in oilfield chemistry and related petroleum engineering scenarios, where their designable network structures and stimuli-responsive rheology can be leveraged for profile control, water shutoff, and other conformance applications. However, compared with these predominantly single-phase or non-biphasic coordination-gel systems, supramolecular gels intentionally engineered in oil–water biphasic media for fracture sealing (lost circulation control) remain much less investigated. More importantly, to the best of our knowledge, no field-oriented plugging system has been systematically reported for lost circulation control in oil-based drilling operations. Such a system would use metal–ligand coordination as the primary gelation motif while also exploiting an oil–water biphasic supramolecular framework. This gap motivates the present work on an oil–water biphasic metal–organic supramolecular gel (MOSG) designed for OBDF lost circulation control.

Based on the principles of metal–organic supramolecular chemistry, this study develops an oil–water biphasic metal–organic supramolecular gel (MOSG) system for wellbore loss control in oil-based drilling fluids. MOSG is generated by mixing oil-phase and aqueous gelation solutions, and its three-dimensional network arises from two synergistic mechanisms: (i) a coordination-driven metal–organic network formed between metal ions and organic ligands; (ii) a surfactant-stabilized biphasic structure in which hydrogen bonding and hydrophobic association build an interconnected soft-matter framework. Meanwhile, the optimized MOSG exhibits rapid gelation, favorable rheological properties, high thermal stability, strong shear resistance, and excellent fracture-sealing performance.

## 2. Results and Discussion

### 2.1. Preparation and Single-Component Optimization of MOSG

#### 2.1.1. Preparation and Field Deployment Methodology of MOSG

The supramolecular gel system developed in this study was mainly composed of industrial diesel, deionized water, the gelling agent phenolic ether phosphate ester (TXP-4), the crosslinker NaAlO_2_, and the emulsifier Span 85. Under the solubilization effect of Span 85, TXP-4 was dissolved in diesel to form the oil-phase gelling solution (OPGS), whereas NaAlO_2_ was dissolved in water to produce an alkaline aqueous gelling solution (AGS) containing aluminate-related species. When OPGS and AGS are mixed, Span 85 reduces the oil–water interfacial tension and promotes a stable oil–water dispersed structure, leading to localized enrichment of the polar headgroups of TXP-4 and Al(III) aluminate/hydrolyzed aluminum species in the interfacial layer. At the interface, the phosphate headgroup of TXP-4 can deprotonate. The phosphate oxygen atoms then serve as coordination sites for ligand exchange with Al(III) species. This process forms P–O–Al bonds and creates bridging crosslinking junctions. Meanwhile, multiple hydrogen-bonding interactions among P=O/phenoxy ether oxygens, water molecules, and aluminum hydroxyl species further stabilize the interfacial structure. As these coordination crosslinking junctions continuously form and expand, microscopic crosslinked clusters gradually percolate to generate a continuous three-dimensional network. Ultimately, the synergistic effects of P–O–Al coordination crosslinking, interfacial hydrophobic aggregation, and the hydrogen-bonding network drive the formation of a macroscopic continuous 3D network, yielding an oil–water biphasic metal–organic supramolecular gel (MOSG). The hydrolysis, neutralization, and coordination reactions involved in the system are as follows:

AlO_2_^−^ + 2H_2_O ⇌ [Al(OH)_4_]^−^, (1)

R_2_HPO_4_ ⇌ R_2_PO_4_^−^ + H^+^, (2)

[Al(OH)_4_]^−^ + (4 − x)H^+^ ⇌ Al(OH)_x_^(3−x)+^ + (4 − x)H_2_O, (3)

Al(OH)_x_^(3−x)+^ + yR_2_PO_4_^−^ ⇌ Al(OH)_x_ (R_2_PO_4_)_y_^(3−x−y)^, (4)

Al^3+^ + 3OH^−^ ⇌ Al(OH)_3_ (s), (5)

where R represents an alkyl group bearing a phenoxy ether moiety; x = 1, 2, or 3, and y ≈ 3 − x. Here, y is the number of phosphate ester groups coordinated to each Al center. The remaining coordination sites are occupied by OH^−^ and/or H_2_O [33].

It should be emphasized that Equations (3)–(5) capture the core reaction pathway governing gelation in this system, namely the pH-dependent transformation of Al species, the coordination crosslinking with phosphate ester ligands, and the competing hydrolysis/precipitation side reaction under extreme conditions. Specifically, Equations (3)–(5) describe the speciation of aluminum as a function of local acid–base environment and its competition between forming soluble coordination-active crosslinkers and insoluble hydrolysis byproducts. Starting from the tetrahydroxoaluminate anion [Al(OH)_4_]^−^ generated in Equation (1), Equation (3) represents stepwise protonation (partial neutralization) by available H^+^, producing a family of hydrolyzed Al(III) species, Al(OH)_x_^(3−x)+^ (x = 1–3), with concomitant water formation. Importantly, these partially hydrolyzed Al centers retain labile coordination sites (occupied by OH^−^/H_2_O) and thus act as “reactive” metal nodes. Equation (4) then describes the key ligand-exchange/coordination step: deprotonated phosphate ester anions (R_2_PO_4_^−^, from Equation (2)) coordinate to the hydrolyzed Al(III) species, replacing some OH^−^/H_2_O ligands and generating mixed hydroxo–phosphate aluminum complexes. Because each Al center can bind multiple phosphate groups (with y ≈ 3 − x), these complexes function as multifunctional junctions that bridge neighboring TXP-4 headgroups at the oil–water interface, enabling the growth and percolation of a three-dimensional network through P–O–Al linkages. Finally, Equation (5) describes the limiting case under stronger hydrolysis/over-neutralization (or locally high OH^−^ activity), where Al(III) converts to insoluble Al(OH)_3_ (s). This precipitation pathway competes with coordination crosslinking. Moderate hydrolysis favors soluble or partially hydrolyzed Al species that participate in Equation (4) and promote gelation. In contrast, excessive hydrolysis drives Al(OH)_3_ precipitation, reducing the availability of coordination-active Al centers and producing inorganic-rich byproducts instead of load-bearing network junctions. In drilling-engineering practice, MOSG is intended to be applied as an independent lost circulation treatment fluid rather than as an additive to the base mud. The system is typically implemented via a dual-liquid injection method, in which the oil-phase gelling solution (OPGS) and aqueous gelling solution (AGS) are prepared separately and pumped into the wellbore through two streams (or sequential slugs). The two solutions are mixed within the wellbore by flow-induced mixing (and/or inline mixing prior to downhole entry), forming a pumpable mixture that can be transported to the loss zone and penetrate into fractures driven by the pressure differential.

After entering the fracture space, MOSG undergoes rapid gelation and structural development, generating a viscoelastic supramolecular network that progressively increases viscosity, builds elastic strength (G′), and forms a continuous, deformable plug. The sealing performance is governed by the coupled effects of (i) gelation kinetics under downhole temperature and residence time, (ii) shear history during pumping through tubulars, and (iii) viscoelastic deformation/recovery of the gel under differential pressure and fracture confinement, which together determine fracture filling, resistance to washout, and ultimate sealing integrity.

#### 2.1.2. Emulsifier Concentration

**Figure 1:**
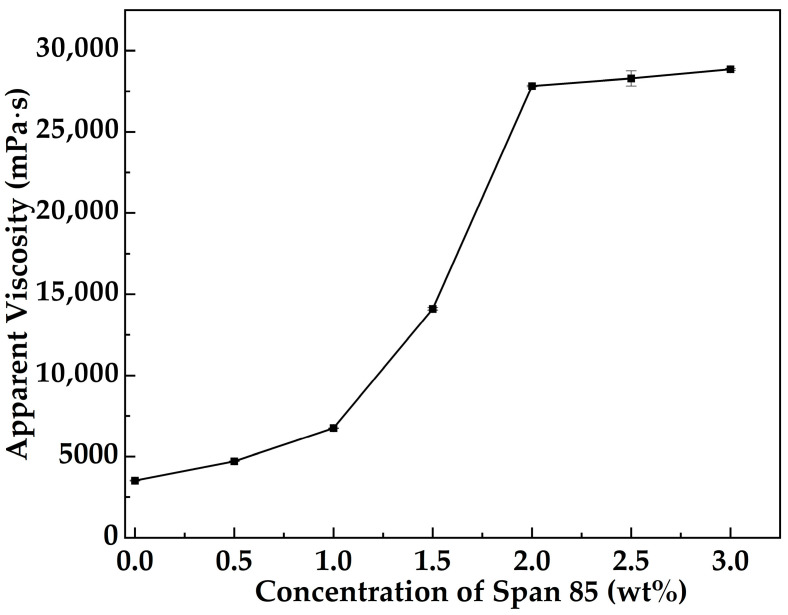
Apparent viscosity of MOSG as a function of Span 85 concentration at 30 °C with a volume ratio of the oil-phase to the aqueous gelling solution of 10:3, 10.0 wt% TXP-4, and 4.0 wt% NaAlO_2_.

Figure 1 illustrates the variation in the apparent viscosity of MOSG as a function of Span 85 concentration at 30 °C, with a fixed oil- to aqueous-phase gelling solution volume ratio of 10:3 and TXP-4 and NaAlO_2_ concentrations maintained at 10.0 wt% and 4.0 wt%, respectively. It can be observed that the addition of Span 85 significantly enhances the gelation performance of the system, and the apparent viscosity stabilizes when the concentration reaches 2.0 wt%. With increasing Span 85 concentration, its emulsification effect on the oil-phase gelling solution is strengthened, facilitating the coordination of Al^3+^ ions from NaAlO_2_ with TXP-4 to form a stable three-dimensional network. Moreover, higher concentrations of Span 85 enhance hydrogen bonding interactions within the system [34]. These multi-point interactions promote further extension and interconnection of the 3D network, ultimately constructing a high-viscosity supramolecular gel.

#### 2.1.3. Oil- to Aqueous-Phase Volume Ratio

**Figure 2:**
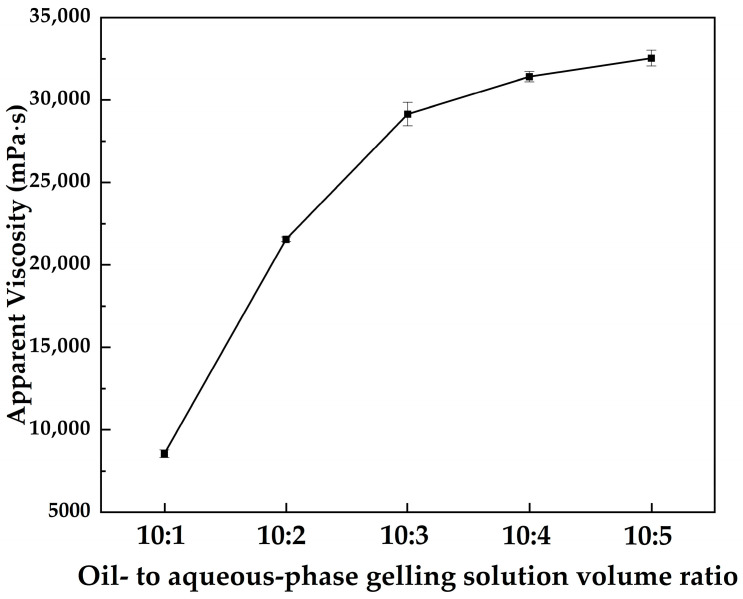
Apparent viscosity of MOSG as a function of the oil- to aqueous-phase gelling solution volume ratio at 30 °C, with 10.0 wt% TXP-4, 4.0 wt% NaAlO_2_, and 2.0 wt% Span 85.

Figure 2 shows the variation in the apparent viscosity of the MOSG system with decreasing volume ratio of the oil-phase to the aqueous gelling solution at 30 °C, where the concentrations of TXP-4, NaAlO_2_, and Span 85 were fixed at 10.0 wt%, 4.0 wt%, and 2.0 wt%, respectively. It can be observed that the apparent viscosity of MOSG increases rapidly with increasing proportion of the aqueous gelling solution and then tends to stabilize. The aqueous gelling solution acts as a polar dispersed phase and participates in the hydrolysis and coordination reactions between the phosphoric ester and sodium aluminate. On the one hand, a higher aqueous fraction reinforces the hydrogen-bonding network and electrostatic interactions in the system; On the other hand, increasing the water content generates more hydrolyzed aluminum hydroxide species. These species provide abundant metal centers for P–O–Al coordination and network crosslinking. As a result, a denser and more continuous metal–organic supramolecular network forms, leading to a pronounced increase in viscosity. When the aqueous fraction is further increased, the oil–water interface becomes progressively stabilized and the emulsion structure and gel network approach equilibrium, so that the apparent viscosity tends toward a plateau. At this stage, the additional water is mainly accommodated within the pores and continuous domains of the pre-existing network, contributing little to further strengthening of the overall structure.

#### 2.1.4. Concentrations of Gelling Agent and Crosslinker

**Figure 3:**
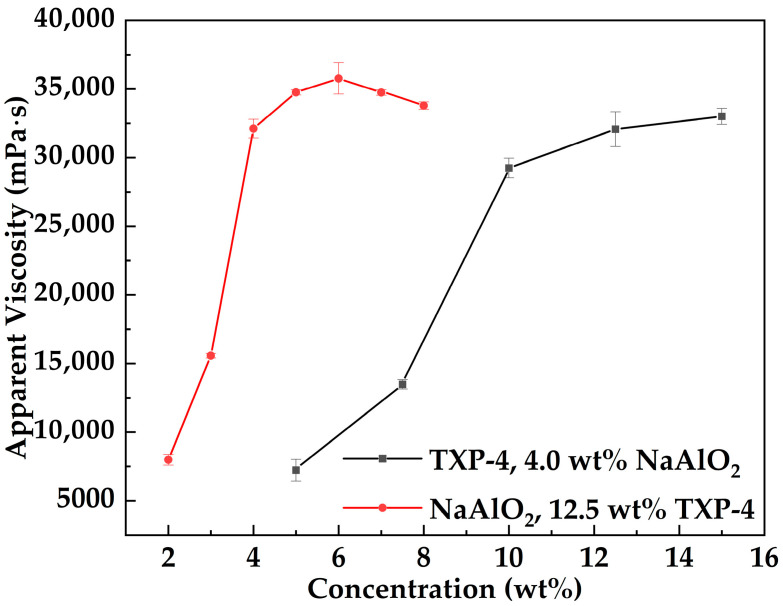
Variation in the apparent viscosity of the MOSG system with NaAlO_2_ (red line) and TXP-4 (black line) concentrations at 30 °C, with a fixed volume ratio of the oil-phase to the aqueous gelling solution of 10:3 and 2.0 wt% Span 85.

Figure 3 presents the variation in apparent viscosity of the MOSG system as a function of NaAlO_2_ and TXP-4 concentrations at 30 °C, with a fixed volume ratio of the oil-phase to the aqueous gelling solution of 10:3 and 2.0 wt% Span 85. It can be observed that the apparent viscosity of MOSG increases rapidly with increasing NaAlO_2_ concentration, but shows a slight decline when the NaAlO_2_ concentration exceeds 6.0 wt%. In contrast, the viscosity increases rapidly with TXP-4 concentration and then gradually reaches a plateau, attaining 32,071 mPa·s at 12.5 wt% TXP-4.

At low concentrations of NaAlO_2_ and TXP-4, the number of reactive and self-assembling functional groups in the system increases rapidly, promoting coordination between phosphate esters and aluminate ions as well as the formation of hydrogen bonds. These interactions accelerate the construction of the supramolecular network, resulting in a marked increase in viscosity. As the concentrations continue to rise, the available crosslinking sites approach saturation, and the network structure becomes more complete. Consequently, further addition of gelling agent or crosslinker has little effect on network density, leading to a plateau in viscosity. Notably, when NaAlO_2_ is excessive (>6 wt%), the increased alkalinity may shift dissolved aluminum toward more stable and less reactive aluminate species (e.g., [Al(OH)_4_]^−^) [35]. Meanwhile, a higher pH may intensify OH^−^ competition for coordination sites, reducing the effective fraction of exchangeable and coordinatively active hydrolyzed Al species [36] and thereby weakening the P–O–Al bridging crosslinking efficiency. In addition, excessive aluminum sources may induce localized enrichment or aggregation of inorganic phases. These rigid inorganic domains are difficult to effectively integrate into the continuous three-dimensional framework network. As a result of these combined effects, the apparent viscosity of MOSG shows a certain degree of decline.

The single-component optimization experiments of the MOSG system indicate that, considering both economic efficiency and gelation performance, the optimal formulation is achieved when the oil-phase to the aqueous gelling solution volume ratio is 10:3, the Span 85 concentration is 2.0 wt%, the NaAlO_2_ concentration is 5.0 wt%, and the TXP-4 concentration is 12.5 wt%. Under these conditions, the MOSG system exhibits excellent gelation properties and structural stability. Therefore, all subsequent studies on the effects of pH, temperature, and shear behavior were carried out using this optimized formulation, so that the structural characterization and plugging performance tests are representative of the practical operating conditions of MOSG in engineering applications.

### 2.2. Analysis of Factors Influencing the Gelation Performance of MOSG

#### 2.2.1. Effect of Reaction Time and Temperature

**Figure 4:**
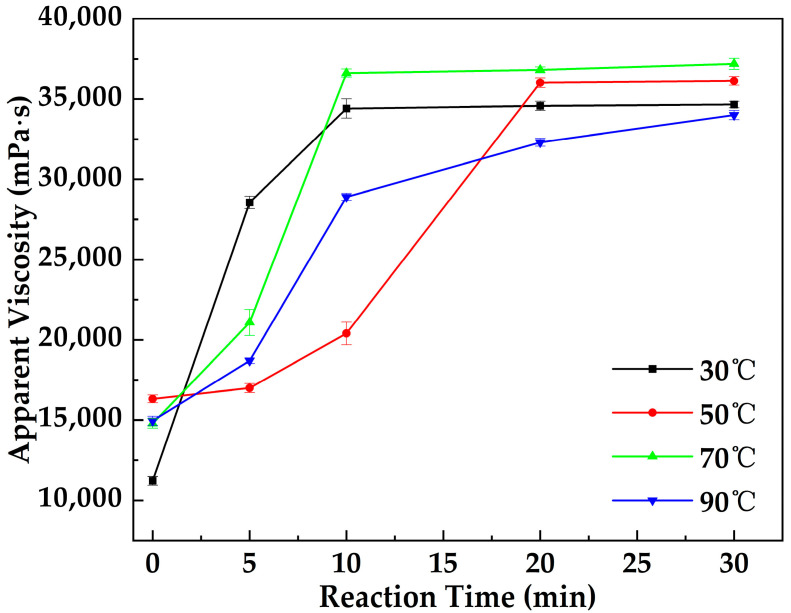
Variation in the apparent viscosity of MOSG with reaction time under different temperature conditions.

Figure 4 illustrates the variation in the apparent viscosity of the MOSG system with reaction time under different temperature conditions. As the reaction time increased, the apparent viscosity of the system gradually rose at all temperatures, indicating the continuous formation and strengthening of the internal gel network structure. At 30 °C, the viscosity reached a stable value after approximately 10 min, suggesting that the gel network was nearly completed. At 50 °C, stabilization occurred around 20 min, whereas at 70 °C, the system again reached equilibrium within about 10 min. In contrast, at 90 °C, the viscosity continued to increase slightly even after 20 min, implying that the molecular motion and reaction rates were higher at elevated temperatures, and the condensation and self-assembly between phosphate esters and aluminate species were still ongoing. Overall, increasing temperature accelerates the gelation process; however, excessively high temperatures may alter the reaction kinetics or compromise the structural stability of the system.

#### 2.2.2. Effect of pH

**Figure 5:**
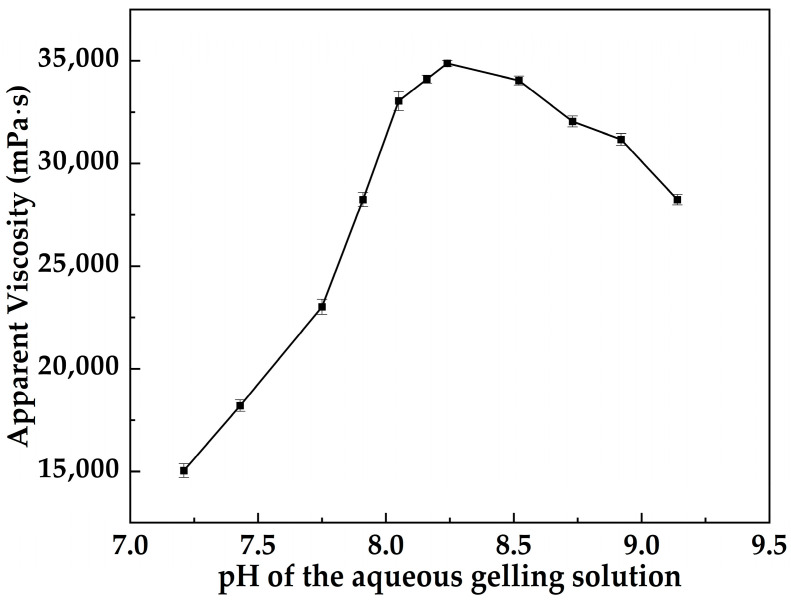
Effect of pH of the aqueous gelling solution on the apparent viscosity of the MOSG system.

Figure 5 shows that the apparent viscosity of MOSG is strongly pH-dependent. As pH of the aqueous gelling solution increases from 7.21 to 8.24, the apparent viscosity rises from 15,042 to 34,874 mPa·s, indicating progressive strengthening of the three-dimensional gel network. Further increasing pH to 8.52 and 9.14 leads to a gradual viscosity decrease, suggesting that excessively alkaline conditions weaken the effective cross-linking structure. Overall, MOSG exhibits an optimum gelation window in the mildly alkaline range of pH ≈ 8.10–8.3.

**Figure 6:**
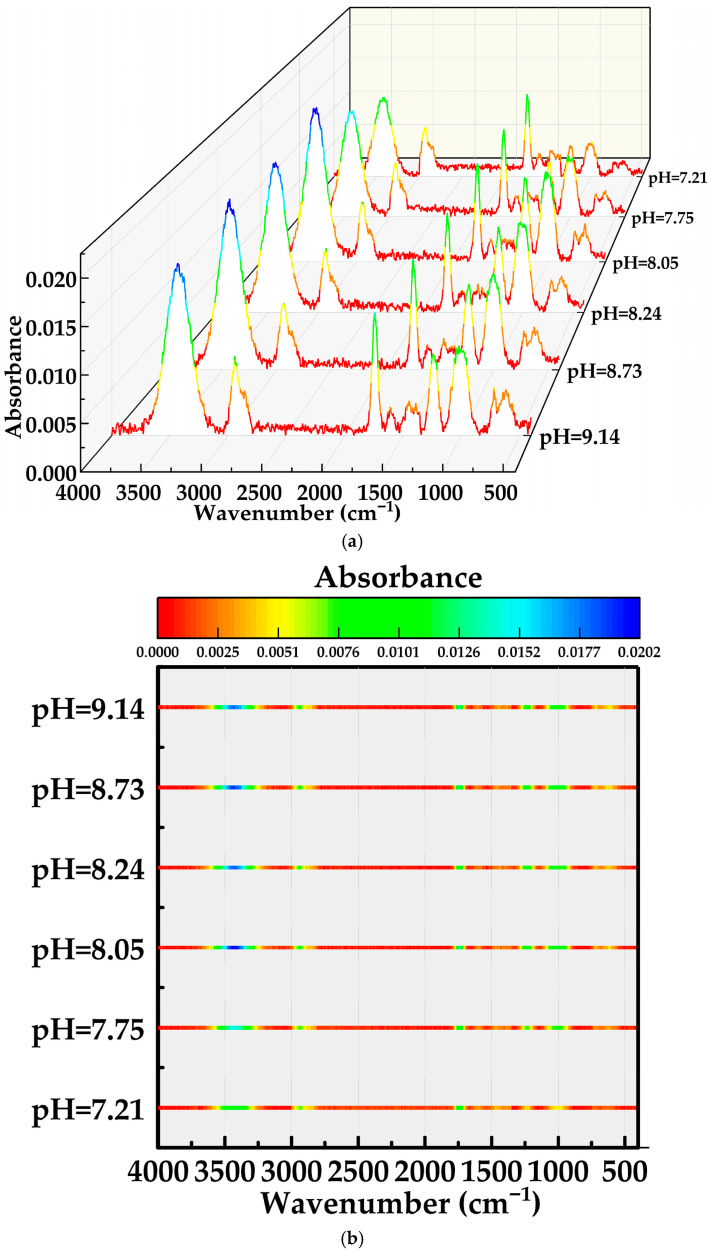
C–H–normalized FTIR spectra of MOSGs at different pH values of the aqueous gelling solution: (**a**) 3D waterfall plot; (**b**) corresponding top-view color map of (**a**).

The FTIR maps in Figure 6a,b provide structural evidence for this pH response. Detailed assignments of peak positions and corresponding bonding modes in the MOSG spectra are provided in Section 2.3.1 (“FTIR Analysis”). In the O–H stretching region (3700–3000 cm^−1^), the broad hydrogen-bonded band becomes progressively stronger and slightly broader when pH increases from 7.21 to 8.05–8.24, consistent with the formation of a denser hydrogen-bond network involving phosphoric ester headgroups, Al–OH species and bound water [37,38,39]. At the same time, the H–O–H bending band of free/bulk water around ~1600 cm^−1^ slightly decreases, implying that more water molecules are converted from “free” to “structurally bound” states within the gel network.

More pronounced pH-induced changes appear in the phosphate- and aluminum-related regions. In the 1250–950 cm^−1^ region, assigned to P=O and P–O stretching as well as P–O–Al coordination, the color in Figure 6b clearly intensifies from pH 7.21 to 8.05–8.24, and then becomes slightly weaker at pH 8.73 and 9.14. This trend parallels the viscosity evolution in Figure 5, indicating that the number and/or strength of P–O–Al coordination bonds is the key factor controlling gel strength [37,38,39,40]. At low pH, TXP-4 is partly protonated and the fraction of deprotonated phosphate anions capable of coordinating Al is limited, so only a loose coordination network is formed. Near pH 8.10–8.3, TXP-4 is sufficiently deprotonated while Al species from the NaAlO_2_–Al(OH)_3_ system are mainly present as polymeric hydroxo complexes that can efficiently bridge between neighboring phosphate groups, giving rise to abundant P–O–Al cross-links and a highly connected supramolecular framework [37,38,39,40]. In the 900–460 cm^−1^ region, corresponding to Al–O and Al–O–P skeletal vibrations, the integrated intensity generally increases with pH, confirming the progressive development of aluminum-containing inorganic domains [38,40]. However, at high pH (≥8.73) the P–O–Al band intensity begins to decrease while the Al–O framework band remains relatively strong. This suggests that a growing fraction of Al species exists as more isolated or over-hydrolyzed hydroxide/aluminate units rather than as effective bridging centers. Excess OH^−^ and highly charged aluminate anions may also compete with phosphate for coordination sites and enhance electrostatic repulsion between phosphate chains [40,41]. As a result, although the amount of Al–O species remains high, their ability to form extended, flexible P–O–Al cross-links is reduced, leading to partial disruption or fragmentation of the supramolecular network and the observed drop in viscosity.

Taken together, Figure 5 and Figure 6 demonstrate that pH regulates the balance among deprotonation of the phosphoric ester TXP-4, hydrolysis and speciation of NaAlO_2_–Al(OH)_3_, and the hydrogen-bonding structure of water. Optimal gel performance is achieved in a narrow mildly alkaline window, where P–O–Al coordination cross-linking and O–H⋯O hydrogen bonding act cooperatively to build a dense yet reversible supramolecular network. At lower pH, coordination is insufficient. Outside this window, gelation is compromised. At higher pH, Al species become over-hydrolyzed and less effective. In both cases, the supramolecular gel network is weakened.

#### 2.2.3. Effect of Shearing Intensity

**Figure 7:**
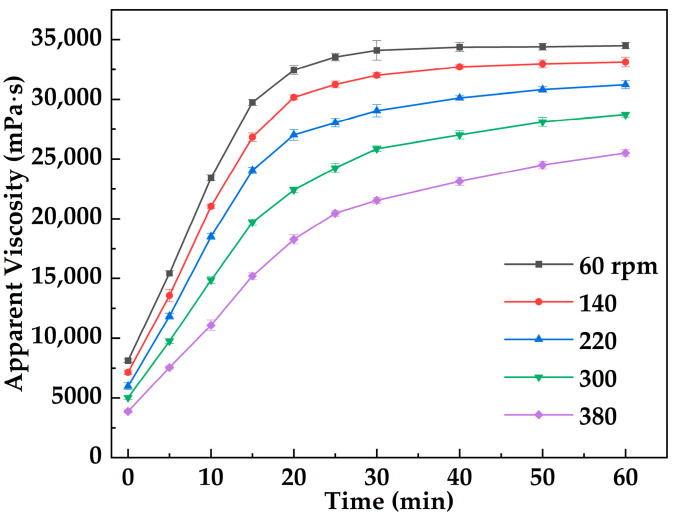
Evolution of MOSG apparent viscosity during gelation at 30 °C under different stirring speeds (60–380 rpm).

Figure 7 illustrates the variation in apparent viscosity of the metal–organic supramolecular gel (MOSG) over time under different imposed stirring (shearing) intensities (60–380 rpm) at 30 °C, applied using an OS60 Pro overhead electric stirrer. Overall, the viscosity of MOSG increased with time under all shear conditions, indicating the continuous formation and strengthening of the gel network. Under lower imposed stirring (60 rpm), the initial viscosity was relatively high (8124 mPa·s) and reached 34,501 mPa·s after 60 min. In contrast, at a higher imposed stirring of 380 rpm, the initial viscosity was only 3874 mPa·s, increasing to 25,514 mPa·s at the end of the test. These results demonstrate that increasing imposed stirring intensity inhibits gelation kinetics and reduces the final viscosity of the system. Shear forces influence both the initial formation and the subsequent evolution of the supramolecular network: mild shear facilitates the assembly of network structures, whereas intense shear disrupts nascent frameworks and delays the development of a continuous three-dimensional structure. The gelation of MOSG relies on metal coordination, hydrogen bonding, and hydrophobic association, exhibiting typical shear sensitivity and self-healing characteristics.

### 2.3. Physicochemical Characterization and Performance of MOSG

#### 2.3.1. FTIR Analysis

**Figure 8:**
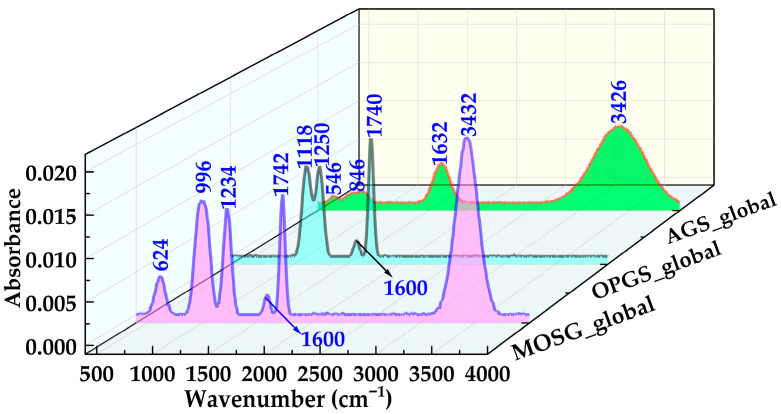
Globally normalized FTIR spectra of the aqueous gelling solution (AGS_global), oil-phase gelling solution (OPGS_global), and supramolecular gel MOSG_global, highlighting the characteristic O–H, P–O and Al–O bands.

Figure 8 compares the globally normalized FTIR spectra of the aqueous gelling solution (AGS), oil-phase gelling solution (OPGS) and the final supramolecular gel (MOSG). The precursor spectra of AGS and OPGS display the typical features of hydrated aluminum hydroxide and organic phosphoric ester systems, respectively, whereas the MOSG spectrum exhibits a number of new bands and band redistributions that cannot be explained by simple superposition. These differences provide spectroscopic evidence consistent with the formation of an organic–inorganic supramolecular network.

For the OPGS, four main bands are resolved at 1740, 1600, 1250 and 1118 cm^−1^. The strong band at 1740 cm^−1^ arises from the C=O stretching of ester groups in TXP-4 and Span 85, and represents the dominant carbonyl signature of the oil phase. The 1600 cm^−1^ band with medium intensity is attributed to C=C/C=O coupled vibrations of unsaturated chains in Span 85. In the fingerprint region, the strong band at 1250 cm^−1^ corresponds mainly to the P=O stretching of the phosphoric ester headgroup, while the 1118 cm^−1^ band is associated with P–O–C and C–O–C stretching, reflecting the ether/ester backbone of TXP-4 and Span 85 [37,38,39]. In contrast, the AGS spectrum is dominated by inorganic features of hydrated Al(OH)_3_. Four major bands are observed at 3426, 1632, 846 and 546 cm^−1^. The broad, intense band at 3426 cm^−1^ originates from O–H stretching of abundant water molecules and hydroxyl groups, indicating the coexistence of free water, bound water and surface –OH. The 1632 cm^−1^ band corresponds to H–O–H bending of water, confirming a strongly hydrated and hydrogen-bonded aluminum hydroxide colloid [37,42]. In the low-frequency region, the bands at 846 and 546 cm^−1^ arise from Al–OH bending/Al–O–Al bridging vibrations and Al–O lattice vibrations, respectively, and indicate the presence of multinuclear, polymerized aluminum–oxygen/hydroxide structures [37,42].

After mixing the two gelling solutions and allowing MOSG to form, the global spectrum in Figure 8 exhibits six main bands centered at 3432, 1742, 1600, 1234, 996 and 624 cm^−1^. Among them, the evolution of the O–H, P-containing and Al–O related bands is most diagnostic. The broad band at 3432 cm^−1^ is still assigned to O–H stretching, but its integrated intensity decreases slightly and the band becomes even broader compared with AGS. This suggests that water and hydroxyl groups no longer interact solely with Al(OH)_3_, but participate in multi-point hydrogen bonding among phosphoric ester headgroups, aluminum hydroxide species and the organic phase. The more heterogeneous O–H environments in MOSG are consistent with a complex supramolecular hydrogen-bond network [37,38,39,42]. The carbonyl band at 1742 cm^−1^ remains strong and appears at essentially the same position as the 1740 cm^−1^ band in OPGS, indicating that the ester backbone of TXP-4 and Span 85 is preserved during gelation and that no significant ester hydrolysis or transesterification occurs. Therefore, the construction of MOSG is most plausibly governed by noncovalent interactions and metal–ligand coordination, rather than by covalent modification of the organic backbone. The most striking changes occur in the phosphate-related fingerprint region. The 1250 cm^−1^ P=O band and the 1118 cm^−1^ P–O–C/C–O–C band of OPGS evolve into a medium band at 1234 cm^−1^ and a new strong band at 996 cm^−1^ in MOSG. The slight red shift of the P=O band (1250 → 1234 cm^−1^) reflects a more electron-deficient environment around phosphorus, consistent with stronger coordination of P=O to Al^3+^ and enhanced hydrogen bonding with water and neighboring –OH/ethoxy groups [37,38,39]. The new band at 996 cm^−1^, which is absent in both AGS and OPGS, is reasonably assigned to P–O–Al stretching and related P–O–Al bridging vibrations. Its high intensity (accounting for roughly one-fifth of the total integrated area) supports the involvement of phosphate–Al interactions, suggesting that TXP-4 phosphoric esters likely coordinate with aluminum hydroxide species to form extended P–O–Al cross-links [37,38,39].

**Figure 9:**
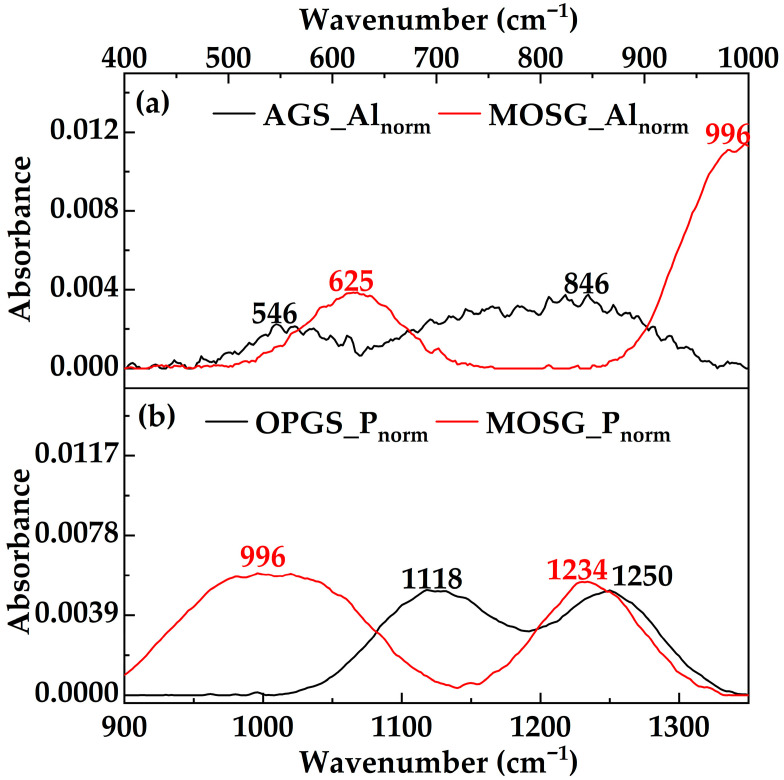
Region-normalized FTIR spectra of MOSG and its precursor gelling solutions in the Al–O and P–O regions: (**a**) Al–O region (400–1000 cm^−1^) spectra of AGS_Al_norm_ and MOSG_Al_norm_; (**b**) P–O region (900–1350 cm^−1^) spectra of OPGS_P_norm_ and MOSG_P_norm_.

Figure 9 further resolves the Al–O and P–O regions by regional normalization. In the Al–O region (Figure 9a), the 846 and 546 cm^−1^ bands of AGS are redistributed into a medium band at 624 cm^−1^ and the composite band around 996 cm^−1^ in MOSG. The 624 cm^−1^ band is stronger and broader than the 546 cm^−1^ band of AGS, indicating that part of the aluminum–oxygen framework has transformed from pure Al–O–Al/Al–OH structures into P–O–Al-bridged aluminum–phosphate networks [37,38,39]. In the P–O region (Figure 9b), the growth of the 996 and 1234 cm^−1^ bands in MOSG relative to OPGS confirms the formation of new coordination-driven cross-links [37,38,39].

Overall, comparison of the globally and regionally normalized spectra of AGS, OPGS and MOSG demonstrates that the oil phase provides P=O and P–O–C functional groups, while the aqueous phase supplies Al–O/Al–OH inorganic motifs. In MOSG, these features are not simply additive. Instead, the emergence of the ~996 cm^−1^ P–O–Al band, the slight red shift of the P=O peak, and the redistribution/broadening of Al–O vibrations collectively suggest metal–phosphate coordination interactions consistent with P–O–Al linkage formation. In parallel, hydrogen bonding and localized inorganic aggregation/precipitation may also contribute to the heterogeneous network features. Together with multi-point hydrogen bonding and hydrophobic association of organic chains, these interactions cooperatively drive the assembly of a three-dimensional supramolecular gel network. The network can be viewed as tri-phase building blocks of “organic phosphoric ester–aluminum hydroxide–water”.

#### 2.3.2. Morphological Characterization

**Figure 10:**
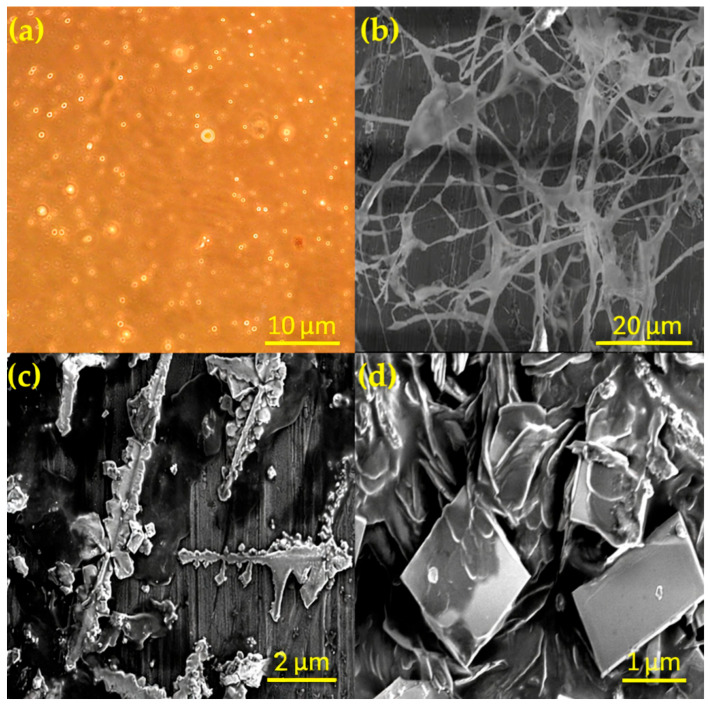
Optical and SEM micrographs of the MOSG system at different magnifications: (**a**) optical microscopy image at 1000× showing dispersed microdomains; (**b**) SEM image at 500× revealing a three-dimensional supramolecular fibrous network; (**c**) SEM image at 7.0k× displaying dendritic crystalline aggregates; (**d**) SEM image at 20.0k× showing well-defined plate-like crystalline structures. Note: SEM images were obtained from freeze-dried samples; therefore, porosity-related features are discussed qualitatively and cross-checked against wet-state optical microscopy.

Figure 10 provides a multiscale view of the MOSG microstructure and provides evidence for a hierarchical gelation mechanism. The optical micrograph in Figure 10a shows uniformly dispersed bright microdomains, which can be interpreted as micron-sized water droplets or interfacial regions encapsulated in the oil phase. At the oil–water interface, the localized enrichment of Al^3+^ species and phosphate-ester headgroups accelerates coordination crosslinking and provides nucleation sites for subsequent network growth. At higher magnification, Figure 10b reveals a continuous three-dimensional fibrous network spanning surface asperities and pore-scale heterogeneities. This morphology indicates cooperative assembly, in which Al^3+^–phosphate coordination and noncovalent interactions (hydrogen bonding, hydrophobic association, and electrostatic attraction) jointly build a mechanically robust supramolecular skeleton. In contrast, the discontinuous dendritic deposits in Figure 10c likely result from localized supersaturation and rapid coordination under restricted diffusion. This interpretation is consistent with diffusion–reaction-controlled precipitation, which commonly produces branched dendritic motifs in inorganic gels and metal-oxide systems [43,44,45]. Figure 10d further displays well-defined plate-like crystalline structures with sharp edges, characteristic of inorganic-rich precipitates such as aluminum phosphate or related hydrolysis products formed under over-coordination or over-hydrolysis conditions; these crystals typically grow as discrete domains within gel matrices and remain structurally separated from the main load-bearing network [45,46,47]. Notably, the presence of these isolated inorganic-rich domains (Figure 10d) supports the interpretation that excessive NaAlO_2_ can induce localized inorganic enrichment/aggregation, rather than contributing to a more integrated load-bearing network.

The coexistence of a continuous supramolecular network and isolated rigid domains suggests a multi-scale structural organization within the MOSG system. The flexible 3D framework enables elasticity, fracture-conforming ability, and self-healing, whereas the localized hard regions may enhance dimensional stability and resistance to deformation under confinement. This hierarchical morphology provides a structural basis for effective fracture bridging and pressure-bearing performance observed in plugging tests. Overall, the microscopic observations support a heterogeneous gelation mechanism, in which coordination-driven assembly yields a dominant supramolecular network while minor inorganic-rich domains arise from localized supersaturation. This hybrid microstructure is intrinsic to metal–organic supramolecular gels and clearly distinct from purely crystalline or purely polymeric systems.

#### 2.3.3. Thermal Stability of MOSG (TGA)

**Figure 11:**
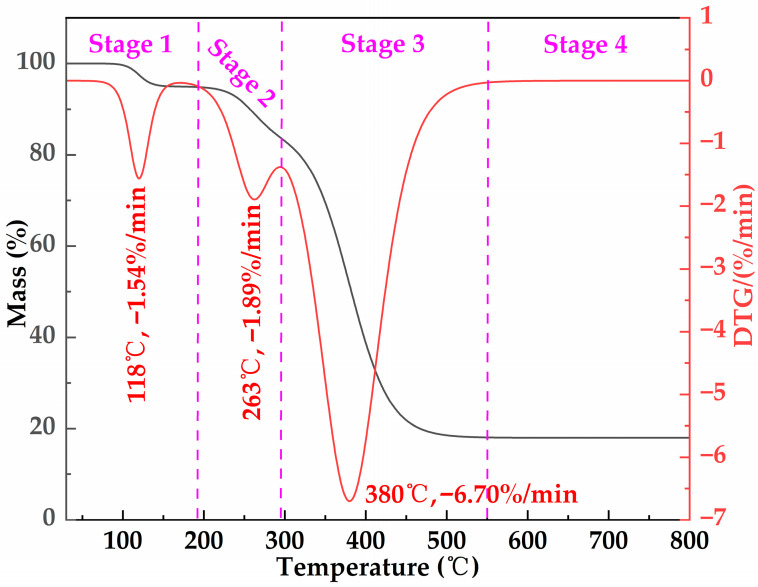
TG and DTG curves of the freeze-dried MOSG under N_2_ at a heating rate of 10 °C/min, showing multi-stage mass-loss events at approximately 118 °C, 263 °C, and 380 °C.

The TG–DTG curves of the freeze-dried MOSG are presented in Figure 11. Under a nitrogen atmosphere, the sample was heated from 30 °C to 800 °C at a heating rate of 10 °C/min, and four stages can be observed. Stage 1 occurs below 193 °C and shows a DTG peak at 118 °C (−1.54%/min), which is mainly attributed to the removal of physically adsorbed water and weakly bound water remaining in the gel network after freeze-drying. Only a slight mass loss is observed in this temperature range, indicating the good low-temperature thermal stability of MOSG. Stage 2 occurs between 193 and 296 °C with a DTG peak at 263 °C (−1.89%/min). This stage may be related to the dehydroxylation transformation of aluminum hydroxyl species and the initial thermal cracking of a small amount of organic/interfacial components. Since the overall mass loss in this stage is small and the DTG peak intensity is relatively low, these results suggest that MOSG mainly undergoes structural relaxation and partial degradation rather than complete decomposition of the network backbone. Stage 3 is the major degradation stage, occurring between 296 and 550 °C, with a pronounced DTG peak at 380 °C (−6.70%/min). This stage mainly corresponds to the thermal cracking/carbonization of organic components such as diesel, Span 85, and TXP-4, accompanied by the destabilization and overall collapse of the network supported by hydrophobic association and Al(III)-phosphate ester coordination. Stage 4 (>550 °C) shows a nearly constant mass, indicating a relatively thermally stable residue mainly composed of inorganic phases and carbonaceous char. From an operational standpoint, the negligible mass loss and the absence of major degradation below ~193 °C indicate intrinsic thermal robustness, suggesting that MOSG is expected to remain structurally stable during typical downhole circulation/placement and sealing stages under elevated-temperature drilling conditions.

#### 2.3.4. Rheological Behavior

**Figure 12:**
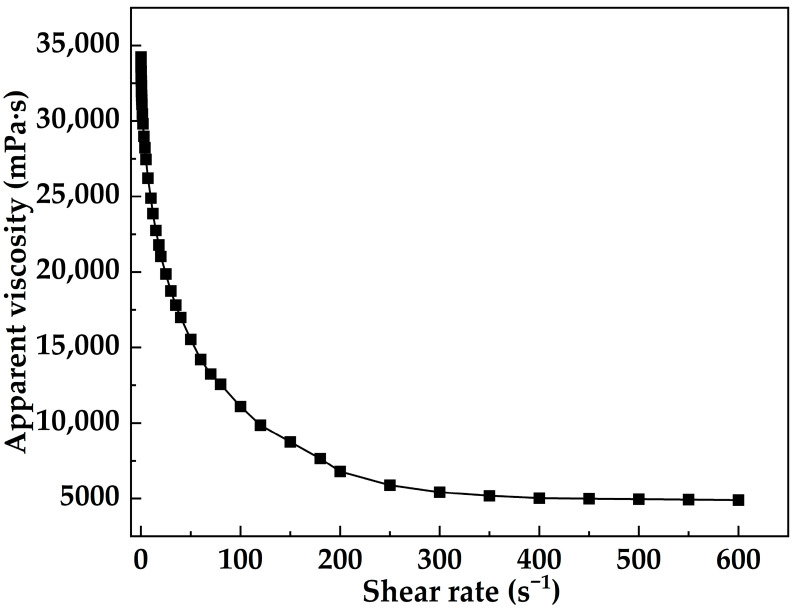
Shear rate dependence of apparent viscosity of MOSG at 30 °C.

**Figure 13:**
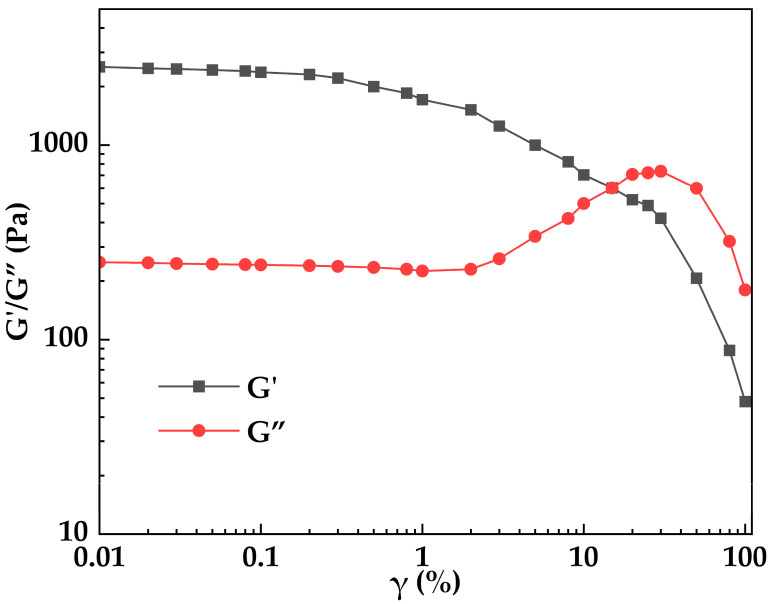
Variation in storage modulus (G′) and loss modulus (G″) of MOSG with shear strain (γ) at 30 °C.

Figure 12 presents the shear-rate sweep results of MOSG at 30 °C. In the low-shear region (0.05–1 s^−1^), the viscosity remained around 34,000 mPa·s, indicating a highly structured network, consistent with the solid-like response (G′ > G″) shown in Figure 13. As the shear rate increased to the intermediate-shear region (1–50 s^−1^), the viscosity decreased sharply, suggesting progressive disruption and reorganization of reversible coordination and hydrogen-bonding interactions. In the high-shear region (50–400 s^−1^), the decrease in viscosity gradually slowed and stabilized at approximately 5000–6000 mPa·s, implying that part of the supramolecular network remained. These results clearly show a shear-thinning transition followed by a high-shear stabilization zone. Such shear-dependent behavior provides an experimental basis for pumpability through the drill string and effective plugging in fractured formations.

Figure 13 illustrates the variation in storage modulus (G′) and loss modulus (G″) of MOSG with shear strain (γ). It can be seen that MOSG is elasticity-dominated and structurally stable in the low-strain regime, whereas in the high-strain regime it yields and becomes viscous (liquid-like) dominated. At low strain (γ ≤ 0.3%), G′ and G″ are nearly constant, with G′ (≈2.2–2.5 kPa) far above G″ (≈0.24–0.25 kPa), indicating a stable, elasticity-dominated network. With increasing strain, G′ decreases while G″ increases, reflecting nonlinear softening and enhanced dissipation. The moduli intersect at γ = 15% (G′ = G″ = 0.602 kPa), marking the yield point. After yielding, G″ reaches a maximum at γ = 30% (0.734 kPa) and then declines, whereas G′ continues to drop, indicating progressive network breakdown. At high strain (γ ≥ 50%), both moduli decrease substantially and the response becomes predominantly liquid-like.

The rheological behavior discussed above can be rationalized by integrating the spectroscopic and microstructural evidence presented in Section 2.3.1 and Section 2.3.2. FTIR analysis reveals the formation of P–O–Al coordination bonds and extensive hydrogen-bonding interactions between phosphate groups, aluminum hydroxide species, and residual water molecules, which together establish a physically crosslinked supramolecular network. The development of such coordination-driven junctions provides the molecular basis for the pronounced elastic response (G′ > G″) observed at low strains. Meanwhile, SEM observations demonstrate a heterogeneous but continuous network composed of interconnected fibrous domains and localized inorganic-rich regions. This microstructure is consistent with the rheological behavior characterized by shear thinning and a well-defined yield point, where deformation induces progressive disruption of noncovalent interactions rather than catastrophic network failure. The combined FTIR, rheological, and morphological evidence therefore supports a gelation mechanism governed by reversible metal–ligand coordination and hydrogen bonding, resulting in a mechanically robust yet deformable supramolecular network suitable for plugging applications under dynamic flow conditions.

#### 2.3.5. Plugging Performance

(1) Seepage-type Loss

In the seepage-loss simulation tests, MOSG exhibited a pressure-bearing capacity of 453.4 kPa in the model permeable formation, which is equivalent to an increase of 2.27 MPa per metre of lost zone. The results indicate that MOSG can effectively crosslink and form a continuous network under elevated-temperature conditions, producing a dense and mechanically robust sealing layer within permeable formations. Consequently, MOSG markedly enhances the resistance of high-porosity/permeability channels to fluid flow and enables reliable plugging of seepage-type loss pathways.

(2) Fracture-type Loss

Table 1 presents the pressure-bearing capacity and pressure gradient of MOSG under different fracture apertures during the fracture-loss tests. MOSG exhibits excellent pressure-bearing and plugging performance under conditions of higher injection pressure and smaller fracture apertures. Both the gel initiation pressure and pressure gradient significantly increase with the injection pressure. For instance, when the injection pressure was raised from 0.50 MPa to 1.5 MPa, the initiation pressure for a fracture aperture of 0.50 mm increased from 42.1 kPa to 624 kPa, and the corresponding pressure gradient rose from 0.842 MPa/m to 12.48 MPa/m. These results indicate that higher injection pressures facilitate deeper penetration of the gel into fractures and enhance its plugging strength. Conversely, at the same injection pressure, both initiation pressure and pressure gradient decreased noticeably with increasing fracture aperture. For example, at 1.0 MPa injection pressure, the initiation pressure dropped from 408 kPa to 298 kPa, and the pressure gradient decreased from 8.16 MPa/m to 5.96 MPa/m as the fracture aperture increased from 0.50 mm to 1.5 mm. This indicates that in narrower fractures, the gel more readily forms a dense sealing layer, whereas wider fractures allow gel dispersion and flow, reducing the plugging efficiency.

#### 2.3.6. Shear Resistance

**Figure 14:**
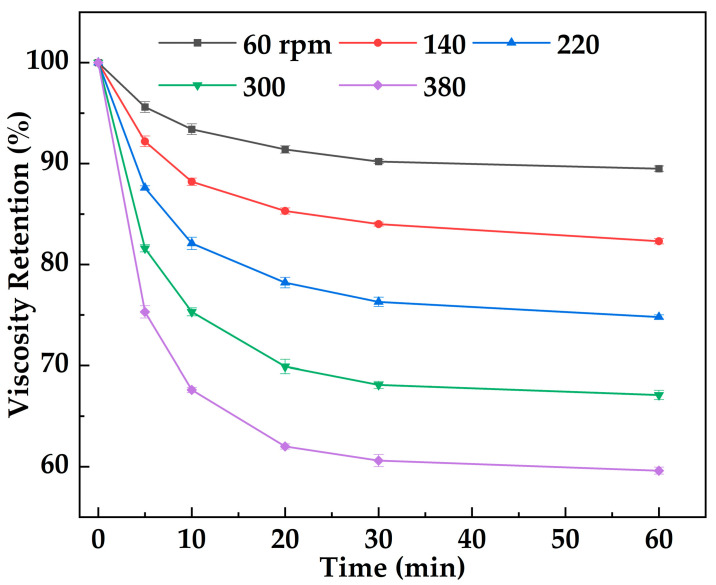
Variation in viscosity retention of MOSG with shear time under different imposed shearing intensities (stirring speed, rpm) at 30 °C.

Figure 14 shows the variation in viscosity retention of MOSG with shearing time under different imposed shearing intensities (stirring speeds, 60–380 rpm). With increasing imposed shearing intensity (60–380 rpm) and shearing time (0–60 min), the viscosity retention first decreases gradually and then tends to stabilize. MOSG exhibits excellent shear resistance and rapid recovery capability. The initial viscosity is 34,512 mPa·s, indicating that MOSG has built a dense three-dimensional network through hydrogen bonding and metal coordination. During the first 10 min of shearing (60 rpm), the viscosity retention decreases, suggesting that part of the network is disrupted, while the overall structure remains largely intact. Even under imposed shearing intensity (380 rpm), the viscosity retention remains above 60%. This behavior is attributed to the dynamic reversibility of noncovalent interactions in the gel (hydrogen bonding, electrostatic interactions, and metal coordination). Shearing can temporarily disrupt the gel network and reduce viscosity. However, once the external force is removed, intermolecular interactions can be re-established and the network can self-assemble and recover, leading to viscosity rebound. This rheological behavior helps MOSG maintain stability and plugging performance under drilling flow conditions.

**Figure 15:**
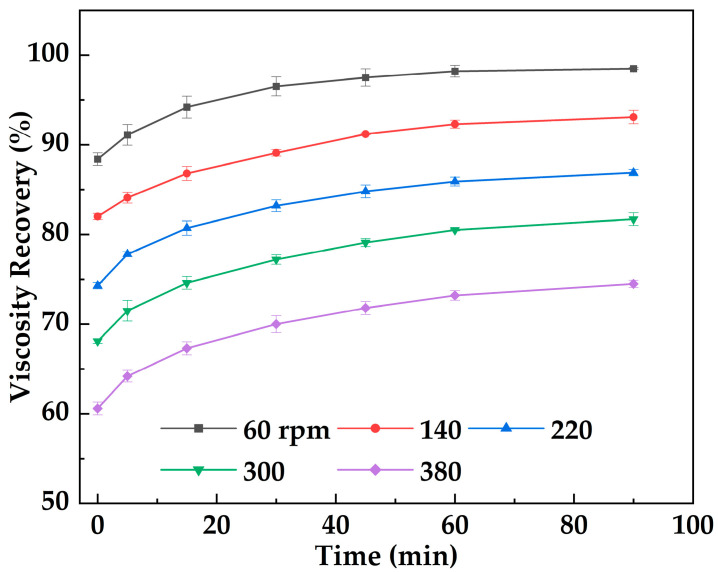
Viscosity recovery of MOSG versus resting time following shear for 30 min under different imposed shearing intensities (stirring speeds, 60–380 rpm) at 30 °C.

Figure 15 shows the variation in viscosity recovery of MOSG with rest time following shear. The results indicate that after gelation and shear disruption (shearing at imposed shearing intensities of 60–380 rpm for 30 min), the viscosity of MOSG gradually recovers during rest, demonstrating an evident self-healing capability. Under a lower imposed shearing intensity (60 rpm), the initial viscosity retention is relatively high (90.2%) and the recovery is rapid, with the viscosity ultimately recovering to 98.5% of the initial value. Under a higher imposed shearing intensity (380 rpm), the initial retention is lower (60.6%), the recovery is slower, and the viscosity finally recovers to 74.5%. Mechanistically, viscosity recovery relies on the dynamically reversible reconstruction of supramolecular noncovalent interactions (e.g., hydrogen bonding, electrostatic interactions, and hydrophobic aggregation) as well as metal–ligand coordination crosslinking. Under high-intensity shear, MOSG undergoes more severe structural disruption and may experience irreversible changes at certain coordination sites or within the emulsified microstructure, thereby limiting the reversible reassembly process.

## 3. Conclusions

To address lost circulation in oil-based drilling fluids, this study investigated an oil–water biphasic metal–organic supramolecular gel (MOSG) system through formulation optimization, parametric evaluation, multiscale characterization, and plugging tests. The main conclusions are as follows.

(1) The optimal MOSG formulation is an oil-phase-to-aqueous-gelling-solution volume ratio of 10:3, 2.0 wt% Span 85, 5.0 wt% NaAlO_2_, and 12.5 wt% TXP-4. Under these conditions, the system exhibits rapid gelation, high apparent viscosity, and good structural stability. Emulsifier, gelling agent, crosslinker contents and the oil–water ratio jointly control the formation, connectivity, and density of the supramolecular network.

(2) Gelation of MOSG is strongly affected by temperature, pH, and shear history. Moderate heating accelerates gel-network development, whereas excessively high temperatures can weaken structural integrity. The system shows optimal gelation and three-dimensional network stability under mildly alkaline conditions of the aqueous gelling solution (pH ≈ 8.10–8.30). Low shear (lower imposed stirring) promotes network assembly, while high shear (higher imposed stirring) disrupts nascent structures and delays the formation of a continuous network.

(3) Spectroscopic, microscopic, and thermal analyses indicate that the MOSG network is built by P–O–Al coordination between phosphate ester headgroups and aluminum hydroxide species, assisted by hydrogen bonding and hydrophobic association in the oil–water biphasic system. The resulting hierarchical microstructure, combining a continuous supramolecular network with localized inorganic-rich domains, provides a robust yet deformable framework that underlies the observed viscoelasticity, shear resistance, and self-healing behavior.

(4) The MOSG system displays good rheological stability, shear-thinning behavior, and thixotropic self-recovery, maintaining a stable three-dimensional network at low strain and partial integrity under high shear. Plugging tests show that MOSG effectively seals both seepage-type and fracture-type losses, and that higher injection pressures and narrower fractures enhance penetration and sealing strength. Even under intense shear, viscosity retention remains above 60% and the gel still shows clear self-healing, supporting its use as an adaptive gel-based lost circulation material in complex reservoirs.

## 4. Materials and Methods

### 4.1. Experiment Materials

Span 85, sodium aluminate (NaAlO_2_), hydrochloric acid (HCl), and sodium hydroxide (NaOH) were of analytical grade and purchased from Sinopharm Chemical Reagent Co., Ltd. (Shanghai, China). Phenolic ether phosphate ester (TXP-4, ≥99%) was supplied by Hai’an Petroleum Chemical Plant (Hai’an, China). Industrial diesel (industrial grade) was obtained from Exxon (Xiamen) Petrochemical Co., Ltd. (Xiamen, China).

### 4.2. Experiment Instruments

Electronic analytical balance (Shanghai Precision Scientific Instrument Co., Ltd., Shanghai, China); Brookfield DV2 Plus-LV viscometer (Brookfield Engineering Laboratories, Inc., Middleboro, MA, USA); OS60 Pro overhead electric stirrer (Shanghai Kexing Instrument Co., Ltd., Shanghai, China); HH-M6 thermostatic water bath (Jiangsu Xinchunlan Scientific Instrument Co., Ltd., Changzhou, China); High-temperature roller oven (Qingdao Xinruide Petroleum Instrument Co., Ltd., Qingdao, China); Polarized optical microscope (CX40P, Ningbo Sunny Instruments Co., Ltd., Ningbo, China); Scanning electron microscope (JSM-6610Lv, JEOL Ltd., Tokyo, Japan); Thermogravimetric analyzer (STA 449F3 Jupiter, NETZSCH-Gerätebau GmbH, Selb, Germany); Fourier-transform infrared spectrometer (Nicolet iS10, Thermo Fisher Scientific, Madison, WI, USA); PHSJ-3F pH meter (Shanghai INESA Scientific Instrument Co., Ltd., Shanghai, China); MCR302 rotational rheometer (Anton Paar, Shanghai, China); and Self-designed plugging test apparatus.

### 4.3. Single-Component Optimization of MOSG

Formulation optimization was conducted using a single-factor (one-variable-at-a-time) strategy. In each set of experiments, only one formulation parameter (Span 85 concentration, oil-to-aqueous phase volume ratio, TXP-4 concentration, or NaAlO_2_ concentration) was varied while the other parameters were kept constant, and the resulting formulations were evaluated after standing for 1 h at 30 °C.

To optimize the supramolecular gel system (MOSG), the apparent viscosity was selected as the primary evaluation parameter to reflect the formation of the network structure and variations in gelation performance. Viscosity serves as an important macroscopic parameter that characterizes the intermolecular interactions and structural compactness within the gel system. It is highly sensitive to changes in the concentrations of the gelling agent, crosslinker, and emulsifier, thus allowing for rapid screening and comparison of different formulations in terms of their gelation tendency and stability [14,48]. Although this method provides an indirect characterization, it has been widely adopted for preliminary optimization and performance assessment of gel systems. Apparent viscosity was measured using a Brookfield DV2 Plus-LV viscometer (Brookfield Engineering Laboratories, Inc., Middleboro, MA, USA) equipped with an LV-4 spindle (entry code 64) at 30 °C and a constant rotational speed of 12 rpm. After spindle immersion to the marked depth, the spindle was rotated for at least five revolutions (or 1 min, whichever was greater) before recording readings, and the reported values were taken after the display stabilized. Viscosity values are reported as apparent viscosity (mPa·s) at the specified speed (12 rpm). In addition, all viscosity measurements described in Section 4.3, Section 4.4, and Section 4.5 were conducted at least in triplicate, and the associated errors were within acceptable limits.

#### 4.3.1. Effect of Emulsifier Concentration

At 30 °C, the volume ratio of the oil-phase to the aqueous gelling solution was fixed at 10:3, with TXP-4 and NaAlO_2_ concentrations maintained at 10.0 wt% and 4.0 wt%, respectively. Different MOSG formulations were prepared by varying the concentration of Span 85. After standing for 1 h, the apparent viscosity of each system was measured using a Brookfield DV2 Plus-LV viscometer to evaluate the influence of emulsifier concentration on the MOSG network formation.

#### 4.3.2. Effect of the Oil-to-Aqueous-Phase Volume Ratio

At 30 °C, TXP-4 and NaAlO_2_ concentrations were fixed at 10.0 wt% and 4.0 wt%, and Span 85 concentration was set at 2.0 wt%. MOSG systems with different oil-phase-to-aqueous-gelling-solution volume ratios were prepared. After 1 h of standing, the apparent viscosity of each system was measured using a Brookfield DV2 Plus-LV viscometer to investigate the effect of oil–water ratio on MOSG gelation.

#### 4.3.3. Effect of Gelling Agent Concentration

At 30 °C, the volume ratio of the oil-phase to the aqueous gelling solution was fixed at 10:3, and the concentrations of NaAlO_2_ and Span 85 were maintained at 4.0 wt% and 2.0 wt%, respectively. Different MOSG formulations were obtained by varying the TXP-4 concentration in the oil-phase gelling solution. After standing for 1 h, the apparent viscosity of each system was measured using a Brookfield DV2 Plus-LV viscometer to assess the influence of gelling agent concentration on the gelation performance of MOSG.

#### 4.3.4. Effect of Crosslinker Concentration

At 30 °C, the oil- to aqueous-phase gelling solution volume ratio was fixed at 10:3, and the concentrations of TXP-4 and Span 85 were maintained at 12.5 wt% and 2.0 wt%, respectively. MOSG systems with different NaAlO_2_ concentrations in the aqueous phase gelling solution were prepared. After standing for 1 h, the apparent viscosity of each system was measured using a Brookfield DV2 Plus-LV viscometer to evaluate the effect of crosslinker concentration on MOSG gelation.

### 4.4. Factors Influencing the Gelation Performance of MOSG

#### 4.4.1. Reaction Time and Temperature

In field applications, the metal–organic supramolecular gel (MOSG) is typically injected through a dual-liquid method for lost circulation control. The two gelling solutions are mixed within the wellbore to form a gel; therefore, it is necessary to investigate the influence of reaction time on gelation performance. At 30 °C, the oil-phase and aqueous gelling solutions were mixed using an OS60 Pro overhead electric stirrer (Shanghai Kexing Instrument Co., Ltd., Shanghai, China), and the viscosity evolution of the mixed system over time was monitored using a Brookfield DV2 Plus-LV viscometer to evaluate the effect of reaction time on gelation. Similarly, the variation in apparent viscosity of the MOSG system with time was measured at 50 °C, 70 °C, and 90 °C to assess the influence of temperature on gelation behavior.

#### 4.4.2. pH Value

The pH of the aqueous gelling solution was adjusted by adding different amounts of HCl or NaOH, and measured using a PHSJ-3F pH meter (Shanghai INESA Scientific Instrument Co., Ltd., Shanghai, China). The pH-adjusted aqueous gelling solution was then mixed with the oil-phase gelling solution and stirred for 5 min, followed by standing for 1 h to allow the system to stabilize. The apparent viscosity of the mixture was subsequently measured using a Brookfield DV2 Plus-LV viscometer to determine the effect of pH on the gelation performance of the MOSG system. For each pH, the corresponding wet MOSG sample was also analyzed by ATR–FTIR (4000–400 cm^−1^), and the spectra were uniformly processed and area-normalized to the diesel C–H stretching region (3032–2770 cm^−1^), so that band-intensity changes directly reflect the influence of pH on supramolecular structure (see Section S1 of Supporting Information).

#### 4.4.3. Shear Effect

During field application, MOSG is typically injected via a dual-liquid method and undergoes continuous high-shear stress as it flows through the wellbore and tubular strings. At this stage, the gelling system remains in a dynamic balance between gelation reactions and structural development; external shear may disrupt the initially formed supramolecular network, thereby affecting the subsequent gelation rate and the final gel strength. To systematically analyze this effect, the oil-phase and aqueous gelling solutions were pre-sheared separately for 50 min at different stirring speeds (60, 140, 220, 300, and 380 rpm) using an OS60 Pro overhead electric stirrer at 30 °C. In this study, rpm is used to represent the imposed shearing intensity via stirring rather than a true shear rate. The two pre-sheared solutions were then mixed and continuously stirred for 3 h under the same stirring speeds. The apparent viscosity of the MOSG system was measured using a Brookfield DV2 Plus-LV viscometer after various standing times to evaluate the influence of shear on gelation kinetics and gel strength.

### 4.5. Characterization and Performance Evaluation of MOSG

#### 4.5.1. Structural and Morphological Characterization of MOSG

(1) FTIR Analysis

ATR–FTIR spectra of the aqueous gelling solution (AGS), oil-phase gelling solution (OPGS), and the metal–organic supramolecular gel (MOSG) were collected on the FTIR spectrometer (Nicolet iS10, Thermo Fisher Scientific, Madison, WI, USA) equipped with a single-reflection diamond ATR accessory. Characteristic shifts in phosphate and hydroxyl group vibrations were used to evaluate coordination interactions and supramolecular assembly behavior. All measurements were conducted within 4000–400 cm^−1^ at a resolution of 2 cm^−1^ with 32 scans, using air as the background. The same testing conditions were applied for all samples to ensure spectral comparability.

(1) Aqueous gelling solution (AGS)

The AGS (NaAlO_2_–Al(OH)_3_ coexistence system) was tested in its wet-gel state. Excess surface water was gently removed prior to measurement to avoid dominating O–H absorption, while maintaining the intrinsic colloidal structure.

(2) Oil-phase gelling solution (OPGS)

Since diesel serves as the continuous oil phase in OPGS, its ATR–FTIR spectrum was first measured as a blank reference. The OPGS spectrum was then acquired under identical conditions. Diesel contributes strong CH_2_/CH_3_ stretching absorptions; therefore, diesel blank subtraction was required to reveal the characteristic bands of TXP-4 and Span 85.

(3) Metal–organic supramolecular gel (MOSG)

MOSG was analyzed directly in its wet-gel form without drying or heating to preserve the native structural environment. Because MOSG also contains diesel, blank diesel subtraction was applied in the same manner as for OPGS to eliminate hydrocarbon background and obtain comparable spectra.

(4) Spectral processing

All spectra were uniformly processed, including baseline correction, smoothing, diesel background subtraction, and normalization. The detailed procedures, normalization windows, equations, and corresponding figures (Figures S4–S6, Tables S2 and S3, and Equations (S4)–(S12)) are provided in the Supporting Information.

(2) Polarized Optical Microscopy and SEM Observation

Fresh MOSG samples were placed on glass slides, covered with coverslips, and observed under bright-field conditions using a polarized optical microscope (CX40P, Ningbo Sunny Instruments Co., Ltd., Ningbo, China) to examine droplet distribution and microphase morphology in the wet state.

For SEM, freeze-dried gel samples (−50 °C, 48 h) were fractured in liquid nitrogen, mounted on conductive carbon tape, and sputter-coated with gold. Imaging was performed using a scanning electron microscope (JSM-6610Lv, JEOL Ltd., Tokyo, Japan) at 1–5 kV and 20 kV to analyze the porous structure, fiber-like networks, and coordination-induced crystalline domains.

#### 4.5.2. Thermogravimetric Analysis (TGA)

Approximately 8 mg of freeze-dried gel sample was placed into an alumina crucible for thermal analysis. Thermogravimetric measurements were carried out using a thermogravimetric analyzer (STA 449F3 Jupiter, NETZSCH-Gerätebau GmbH, Selb, Germany) under a nitrogen atmosphere. The temperature range was set from 30 to 800 °C with a heating rate of 10 °C/min.

#### 4.5.3. Rheological Properties

After placement in fractures, MOSG must simultaneously maintain pumpability during injection/transport and rapidly develop solid-like elasticity to form a stable seal under differential pressure. Therefore, oscillatory rheology (G′, G″) and steady-shear measurements were performed to quantify the viscoelastic transition and shear-thinning behavior of the MOSG system.

Supramolecular gels are typical viscoelastic materials, exhibiting both viscous and elastic characteristics. When an external stress induces deformation, part of the energy is stored as elastic potential energy, while the remainder is dissipated as heat. When the storage modulus (G′) exceeds the loss modulus (G″), the material behaves like a solid and the deformation is recoverable; conversely, when G″ is greater than G′, the material exhibits fluid-like behavior and the deformation is irreversible.

To systematically investigate the viscoelastic response of MOSG after gelation, the storage modulus (G′) and loss modulus (G″) were measured under small-amplitude oscillatory shear using a rotational rheometer. The variations in G’ and G″ with shear strain were recorded to elucidate the energy storage and dissipation behavior of the system. The experimental conditions were as follows: 30 °C, PP50 rotor, oscillation frequency 1.0 Hz, and strain range 0.01–100%. In addition, steady shear measurements were conducted to determine the apparent viscosity as a function of shear rate, allowing analysis of the flow behavior and shear-thinning characteristics of the MOSG system. Steady shear tests were performed at 30 °C with a PP50 rotor, 1.0% strain, and a shear rate range of 0.01–600 s^−1^.

#### 4.5.4. Plugging Performance Test

According to the mechanism of lost circulation, potential loss types can be classified into four categories: seepage loss, fracture loss, cavernous loss, and induced loss [12,49,50]. Among them, cavernous loss is typically mitigated by cement slurry injection, while induced loss can be prevented by controlling the bottom-hole pressure. In contrast, seepage loss and fracture loss are the major challenges frequently encountered when drilling with oil-based fluids. In this work, the plugging performance of the supramolecular gel (MOSG) was evaluated against both seepage-type and fracture-type losses.

(1) Seepage-Loss Simulation

**Figure 16:**
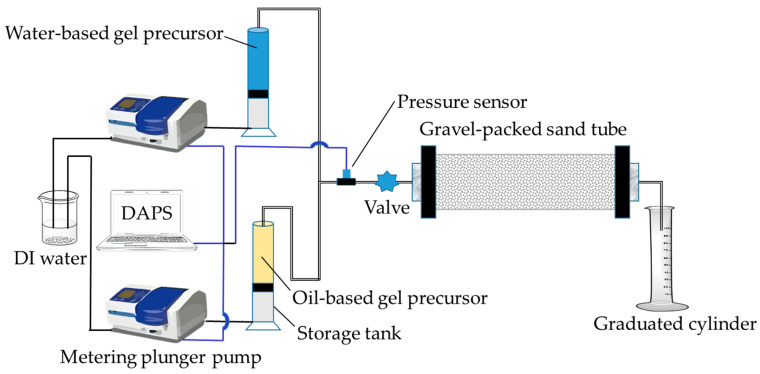
Experimental setup of gel plugging test for seepage-loss simulation. DAPS = Data Acquisition and Processing System.

The plugging performance of MOSG in permeable formations was evaluated using the experimental setup shown in Figure 16. The gravel-packed sand tube (inner diameter: 2.5 cm, length: 20 cm, maximum pressure: 10.0 MPa) was packed with quartz gravel (particle size: 4.75–4.00 mm) to simulate a high-porosity formation. Water-based and oil-based gel precursors were injected into the sand tube using a metering plunger pump at flow rates of 1.00 mL/min and 0.40 mL/min, respectively, until viscous pre-gel effluent appeared at the outlet. The sand tubes containing the injected gels were then aged in an oven at 120 °C for 16 h. Finally, deionized water was injected into the sand tube at 1.00 mL/min, and the pressure recorded at the moment the first water droplet appeared at the outlet was taken as the pressure-bearing capacity of the supramolecular gel in the simulated permeable formation.

(2) Fracture-Loss Simulation

**Figure 17:**
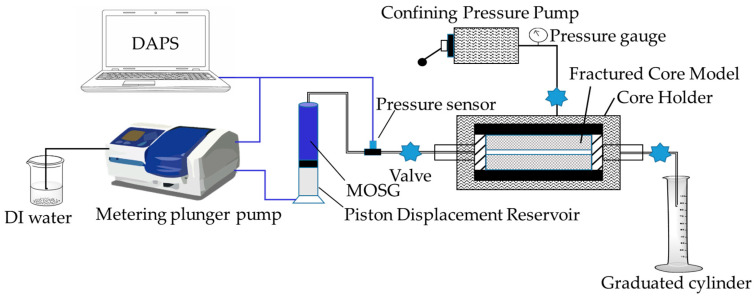
Experimental setup of gel plugging test for fracture-loss simulation. DAPS = Data Acquisition and Processing System.

The experimental setup shown in Figure 17 was used to assess the plugging performance of MOSG in fractured formations. The piston displacement reservoir was filled with the prepared supramolecular gel, and the core holder was set under a confining pressure of 10.0 MPa. The gel was injected into the fractured core model at a flow rate of 0.50 mL/min using a metering plunger pump. Once gel effluent was observed at the outlet, the downstream valve was closed, and gel injection continued until the injection pressure sequentially reached 0.50 MPa, 1.0 MPa, and 1.5 MPa. The core holder was then aged in a roller oven at 120 °C for 16 h. Finally, deionized water was injected into the core holder at a rate of 0.50 mL/min, and the pressure at which the first water droplet appeared at the outlet was recorded as the pressure-bearing capacity of MOSG in the simulated fractured formation.

The fractured core model consisted of two semi-cylindrical steel halves and was used to simulate a single fracture with a length of 2 inches, width of 2 inches, and fracture apertures of 0.5, 1.0, and 1.5 mm, respectively.

#### 4.5.5. Shear Resistance Test

Due to the short gelation time of MOSG, partial or complete gelation may occur while the fluid is still flowing within the drilling string and wellbore. Consequently, the formed gel can be subjected to sustained shear stress during continued pumping/circulation, and may also experience downhole circulation and pressure fluctuations (e.g., transient shear and intermittent resting). Therefore, it is necessary to evaluate the shear resistance of MOSG in terms of its structural stability under prolonged shear and its ability to recover during resting, which are critical for maintaining sealing integrity and preventing washout in field operations. At 30 °C, the supramolecular gel was subjected to different imposed stirring (shearing) intensities (60, 140, 220, 300, and 380 rpm) using an OS60 Pro overhead stirrer, and its viscosity was measured over time with a Brookfield DV2 Plus-LV viscometer. Viscosity retention was defined as the ratio of the gel viscosity after a given shear duration to its initial viscosity, reflecting the gel’s ability to withstand shear. Viscosity recovery was defined as the ratio of the gel viscosity after resting for a certain period following shear to its initial viscosity, indicating the gel’s capacity to restore its structure post-shear. Both parameters were monitored to assess the effect of shear on the gel’s rheological stability.

## Figures and Tables

**Table 1:** Pressure-bearing capacity and pressure gradient of MOSG in fracture-type loss tests under different fracture apertures and injection pressures.

Fracture Aperture (mm)	Injection Pressure (MPa)	Pressure-Bearing Capacity (kPa)	Pressure Gradient (MPa/m)
0.50	0.50	42.1	0.842
	1.0	408	8.16
	1.5	624	12.48
1.0	0.50	35.7	0.714
	1.0	343	6.86
	1.5	475	9.5
1.5	0.50	30.4	0.608
	1.0	298	5.96
	1.5	405	8.10

## Data Availability

The data that support the findings of this study are available from the corresponding author upon reasonable request.

## Supplementary Materials

The following supporting information can be downloaded at: https://www.mdpi.com/article/10.3390/gels12010074/s1, Figure S1: Raw FTIR spectra of MOSGs at different pH values; Figure S2: Uniformly processed FTIR spectra of MOSGs at different pH values following denoising, smoothing, baseline correction, and non-negative trimming; Figure S3: Area-normalized FTIR spectra of MOSGs using the 3032–2770 cm^−1^ C–H region; Figure S4: Raw ATR–FTIR spectra of the samples. OPGS = oil-phase gelling solution; AGS = aqueous gelling solution; MOSG = metal-organic supramolecular gel; Figure S5: ATR–FTIR spectra after baseline correction, denoising, and smoothing. OPGS = oil-phase gelling solution; AGS = aqueous gelling solution; MOSG = metal-organic supramolecular gel; Figure S6: Diesel-subtracted ATR–FTIR spectra of the oil-phase gelling solution (OPGS) and the metal-organic supramolecular gel (MOSG), together with the spectrum of the aqueous gelling solution (AGS); Table S1: Integrated peak areas (3032–2770 cm^−1^) of MOSGs at different pH values; Table S2: Integrated peak areas (3032–2770 cm^−1^) of diesel, OPGS, and MOSG, and the corresponding scaling factors k; Table S3: Integrated absorbance areas used for global, P-region, and Al-region normalization of the OPGS, AGS, and MOSG spectra.

## Abbreviations

The following abbreviations are used in this manuscript:

AGS	Aqueous Gelling Solution
DTG	Derivative Thermogravimetry
FTIR	Fourier Transform Infrared Spectroscopy
HPHT	High-Pressure High-Temperature
LCM	Lost Circulation Material
MOG	Metal–Organic Gel
MOSG	Oil–Water Biphasic Metal–Organic Supramolecular Gel
OBDF	Oil-Based Drilling Fluid
OPGS	Oil-Phase Gelling Solution
SEM	Scanning Electron Microscopy
TG	Thermogravimetry
TGA	Thermogravimetric Analysis
TXP-4	Phenolic Ether Phosphate Ester (Gelling Agent)
