# Supplementary material for: Oil-Water Biphasic Metal-Organic Supramolecular Gel for Lost Circulation Control: Formulation Optimization, Gelation Mechanism, and Plugging Performance
Source: Gels. 2026 Jan 15;12(1):74. doi: 10.3390/gels12010074 (PMC12840760; doi:10.3390/gels12010074)
Supplement: Supplementary file 1 [file gels-12-00074-s001.zip › gels-4055186-supplementary.pdf]

Supporting Information for Oil–Water Biphasic Metal–Organic Supramolecular Gel for Lost Circulation Control: Formulation Optimization, Gelation Mechanism, and Plugging Performance

Qingwang Li [1,2,3], Songlei Li [4], Ye Zhang [1,2,*], Chaogang Chen [5], Xiaochuan Wu [1,2], Menglai Li [1,2], Shubiao Pan [1,2], Junfei Peng [1,2]

1. National Joint Engineering Research Center for Shale Gas Exploration and Development, Chongqing Institute of Geology and Mineral Resources, Chongqing 400042, China; amazingwxl@163.com (Q.L.); lml_90@sina.com (M.L.); hsiaochuanwu@hotmail.com (X.W.); pan_shubiao@163.com (S.P.); pengjunfeihuihui@163.com (J.P.)
2. Key Laboratory of Shale Gas Exploration, Ministry of Land and Resources, Chongqing Institute of Geology and Mineral Resources, Chongqing 400042, China
3. China North Chemical Research Academy Group Co., Ltd., Beijing 100080, China;
4. Downhole Services Company, BHDC, Renqiu 062552, China; lisonglei126@126.com
5. Sichuan Energy Investment Oil and Gas Exploration and Development Co., Ltd, Chengdu 610000, China; chenchaogang2008@126.com

* Correspondence: zhangye_sg@vip.163.com

1. FTIR Data Processing for pH-Dependent MOSG Gelation

To ensure the comparability of ATR–FTIR spectra across different systems and pH conditions, all raw spectra were first subjected to uniform denoising, smoothing, baseline correction and non-negative clipping. Subsequently, area normalization was performed using the alkyl C–H absorption band within 3032–2770 cm^{-1} as an internal reference.

1.1 Spectral Denoising, Smoothing, and Baseline Correction

All ATR–FTIR spectra of the metal–organic supramolecular gels (MOSGs) were acquired under the same instrumental settings (4000–400 cm^{-1}; resolution: 2 cm^{-1}; 32 scans), and the unprocessed spectra are displayed in Figure S1. Before quantitative interpretation, the spectra were subjected to a unified preprocessing workflow in Origin. Initially, a Savitzky–Golay smoothing routine (9-point window, second-order polynomial) was applied to gently attenuate high-frequency noise while maintaining the intrinsic band profiles. The smoothed spectra were then manually baseline-corrected to remove residual drift and to compensate for any sloping or curved background.

After spectral smoothing and baseline correction, small negative absorbance values may still appear in the baseline-corrected spectra due to fitting errors and random noise. To prevent these artificial negative signals from being amplified during integration and subsequent analysis, all corrected spectra were subjected to a unified non-negative clipping step. For each wavenumber $\tilde{\nu}$, if the corrected absorbance $A_{corr}(\tilde{\nu})$ was negative, it was set to zero, yielding:

$$A_{clip}(\tilde{\nu}) = \max\left(A_{corr}(\tilde{\nu}),\ 0\right) \tag{S1}$$

This operation only affects very weak signals close to the baseline and does not

change the shape or intensity of well-defined absorption bands. It provides a stable, physically reasonable dataset for the subsequent integration and normalization procedures. The fully processed spectra used for subsequent analysis are shown in Figure S2.

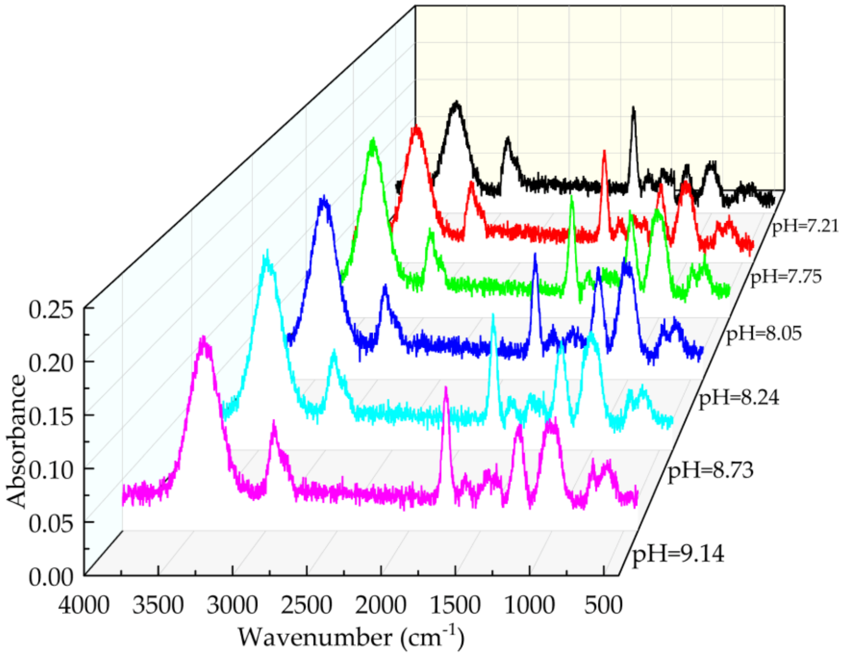

Figure S1. Raw FTIR spectra of MOSGs at different pH values.

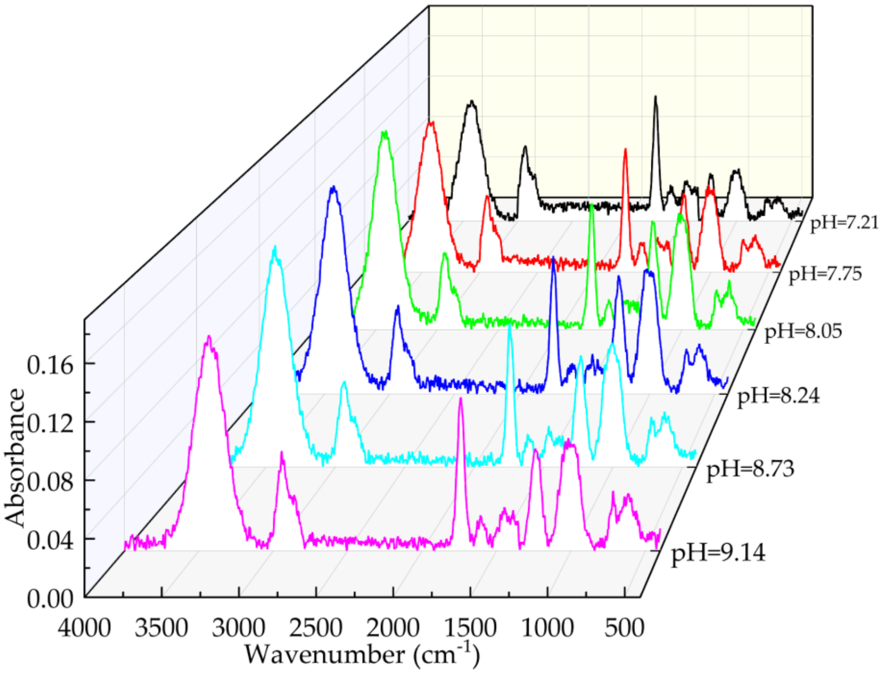

Figure S2. Uniformly processed FTIR spectra of MOSGs at different pH values following denoising, smoothing, baseline correction, and non-negative trimming.

1.2 Normalization Calculations

Based on $A_{clip}(\tilde{\nu})$, a band-area normalization procedure was applied to eliminate global intensity variations between samples arising from differences in ATR contact, sample thickness, or local concentration, rather than from genuine structural changes.

To this end, an internal reference region was selected that (i) corresponds to a chemically invariant component across all samples and (ii) is only weakly affected by pH. In this work, the 3032–2770 cm^{-1} region, corresponding to the aliphatic C–H stretching vibrations of the diesel phase, was chosen as the internal reference band. Since the oil–water ratio and diesel content were kept constant for all pH conditions, the C–H band can be treated as a stable intensity reference.

For each sample *i*, the band area in this region was calculated by integration of the clipped spectrum:

$$S_i = \int_{2770}^{3032} A_{clip}(\tilde{v})\, d\tilde{v} \qquad \text{(S2)}$$

Table S1 shows the integrated peak areas (3032–2770 cm^{-1}) of MOSGs at different pH values. The entire spectrum of sample i was then scaled by its corresponding reference area S_i to obtain the normalized absorbance:

$$A_{norm}(\tilde{v}) = \frac{A_{clip}(\tilde{v})}{S_i} \qquad \text{(S3)}$$

Table S1. Integrated peak areas (3032–2770 cm^{-1}) of MOSGs at different pH values .

Sample	$S_{i,\ 3032-2770}$
MOSG (pH=7.21)	10.08
MOSG (pH=7.75)	9.43
MOSG (pH=8.05)	8.55
MOSG (pH=8.24)	9.19
MOSG (pH=8.73)	8.59
MOSG (pH=9.14)	8.29

This procedure effectively sets the integrated area of the C–H reference band to unity for all samples, “anchoring” the overall spectral intensity to a common internal standard. As a result, any differences observed in the intensities or integrated areas of the P–O / P–O–Al, Al–O, and O–H bands can be primarily attributed to pH-induced structural changes in the MOSG network, rather than to experimental artefacts such as sample loading or contact variations. This greatly improves the reliability of comparative FTIR analysis across the pH series. The area-normalized FTIR spectra of MOSGs are shown in Figure S3.

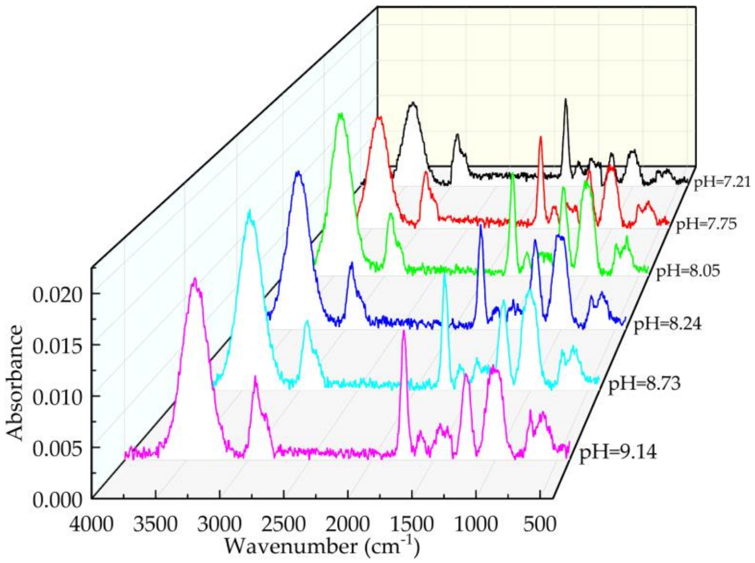

Figure S3. Area-normalized FTIR spectra of MOSGs using the 3032–2770 cm^{-1} C–H region.

2. FTIR Data Processing for MOSG Characterization

2.1. Diesel Background Subtraction

All ATR–FTIR spectra of diesel, the aqueous gelling solution (AGS), the oil-phase gelling solution (OPGS), and the metal–organic supramolecular gel (MOSG) were collected under identical instrumental conditions (spectral range: 4000–400 cm^{-1}; resolution: 2 cm^{-1}; 32 scans). The raw spectra are shown in Figure S4. Prior to further analysis, all spectra were uniformly preprocessed using Origin software. First, slight noise reduction was performed by applying a Savitzky–Golay smoothing filter (9 data points, second-order polynomial) to suppress high-frequency noise while preserving the original band shapes. Subsequently, baseline correction was carried out manually to remove residual signal drift and eliminate any sloping or curved baselines. The resulting preprocessed spectra are presented in Figure S5.

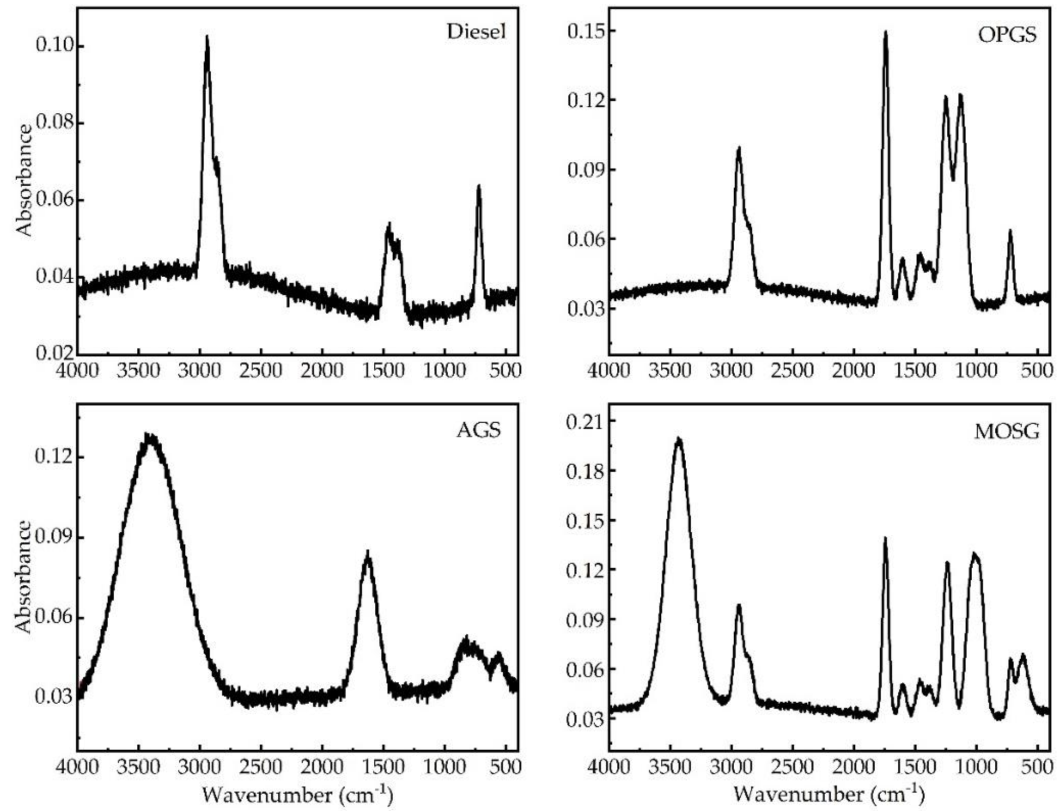

Figure S4. Raw ATR–FTIR spectra of the samples. OPGS = oil-phase gelling solution; AGS = aqueous gelling solution; MOSG = metal–organic supramolecular gel.

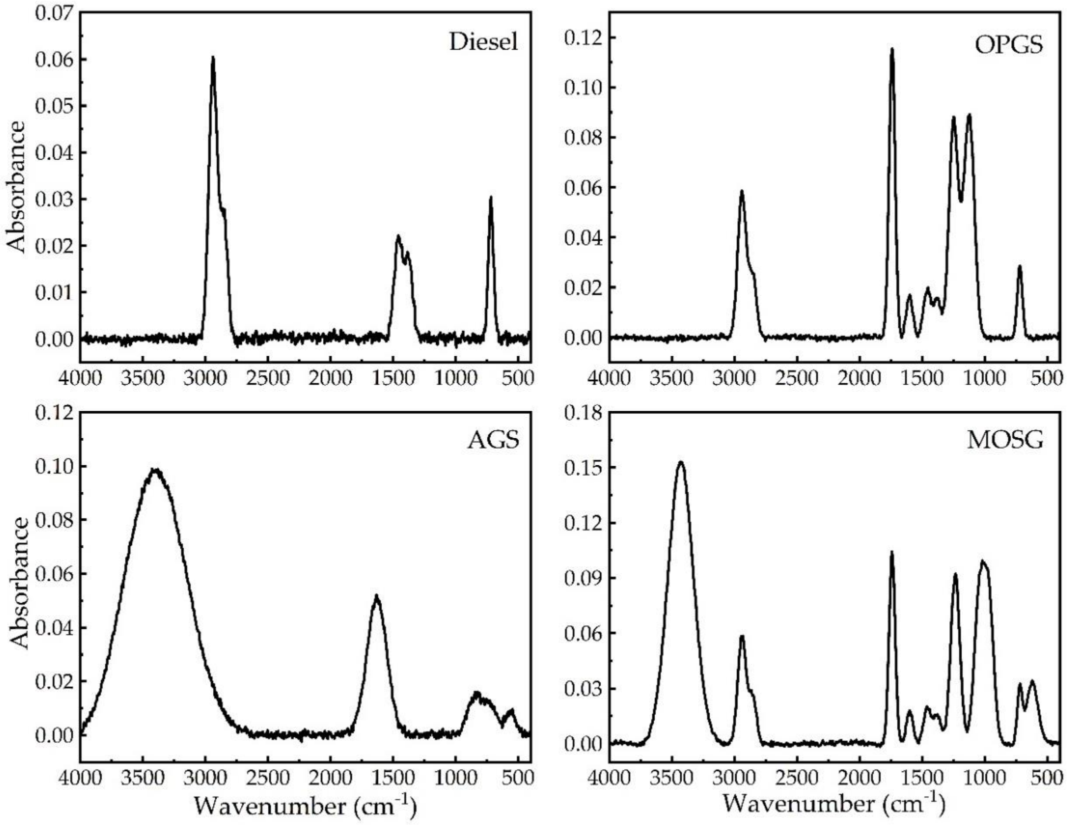

Figure S5. ATR–FTIR spectra after baseline correction, denoising, and smoothing. OPGS = oil-phase gelling solution; AGS = aqueous gelling solution; MOSG = metal–

organic supramolecular gel.

In this study, both OPGS and MOSG used industrial diesel as the continuous oil phase. Consequently, the directly obtained ATR–FTIR spectra contained strong aliphatic C–H stretching absorptions originating from diesel. To isolate the characteristic absorption bands associated with the gelling agent (TXP-4), the emulsifier (Span 85), and their coordination structures, diesel background subtraction was required.

The primary spectral contribution of diesel appears as a single broad envelope of CH_2/CH_3 stretching vibrations in the 3032–2770 cm^{-1} region. To accurately remove this contribution, an appropriate scaling factor k must be determined for the diesel background spectrum. Specifically, using the preprocessed spectral data (Figure S5), the integrated peak areas of diesel, OPGS, and MOSG within the 3032–2770 cm^{-1} region were calculated. The corresponding scaling factor k was then obtained according to Equation S4. The diesel background was then subtracted from each sample spectrum according to Equation S5.

$$k = \frac{S_{sample,\ 3032-2770}}{S_{diesel,\ 3032-2770}} \tag{S4}$$

$$A_{sample}(\tilde{\nu}) = A_{sample}(\tilde{\nu}) - k \cdot A_{diesel}(\tilde{\nu}) \tag{S5}$$

Table S2. Integrated peak areas (3032–2770 cm^{-1}) of diesel, OPGS, and MOSG, and the corresponding scaling factors k.

Sample	$S_{sample,\ 3032-2770}$	k
Diesel	6.79	-
OPGS	6.65	0.979
MOSG (pH=8.16)	6.70	0.987

Because the strong absorption in the 3032–2770 cm^{-1} region originates almost entirely from the aliphatic C–H stretching vibrations of diesel, matching the integrated area of this region between the diesel spectrum and each sample spectrum ensures that the diesel contribution is effectively removed. At the same time, characteristic absorptions associated with the organic backbone of TXP-4 and Span 85, as well as coordination-related features, are preserved. Figure S6 displays the diesel-subtracted ATR–FTIR spectra of OPGS and MOSG, together with the spectrum of the aqueous gelling solution (AGS). These spectra were used for subsequent comparative analysis of the supramolecular structures.

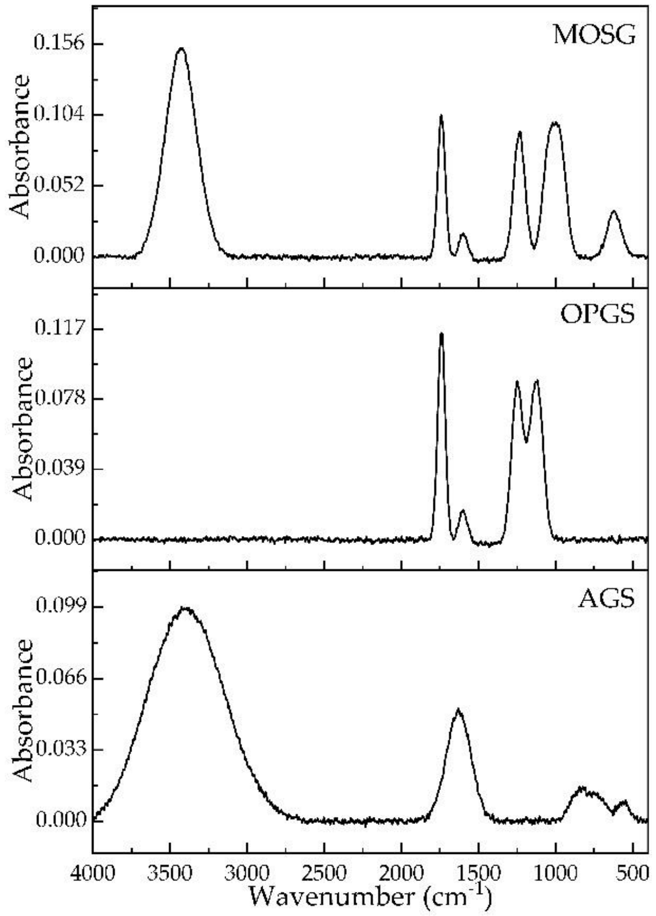

Figure S6. Diesel-subtracted ATR–FTIR spectra of the oil-phase gelling solution (OPGS) and the metal–organic supramolecular gel (MOSG), together with the spectrum of the aqueous gelling solution (AGS).

2.2. Normalization Methods

2.2.1. Selection of Normalization Windows

2.2.1.1. 1400–1800 cm^{-1} (Global Normalization Window)

The region of 1400–1800 cm^{-1} was selected for global normalization of the three spectra (OPGS, AGS, and MOSG) based on the following considerations:

1) All three systems exhibit substantial absorption in this region, and their intensities fall within a comparable magnitude. OPGS and MOSG contain the ester C=O stretching vibration and associated skeletal modes. AGS and MOSG show characteristic H–O–H bending absorption.

2) Compared to the high-frequency O–H stretching region (3600–3000 cm^{-1}), which is dominated almost entirely by the aqueous phase, the 1400–1800 cm^{-1} region better represents the "common spectral backbone" among the three systems, avoiding extreme scaling caused by near-zero absorption in one spectrum.

3) Relative to lower-wavenumber regions (<1000 cm^{-1}), where diesel-phase absorption becomes weak, the 1400–1800 cm^{-1} region provides higher signal-to-noise ratio and a more stable baseline, making the integrated area less sensitive to noise.

For these reasons, the 1400–1800 cm^{-1} interval was chosen as the global normalization window, providing a robust and unbiased intensity reference for the

comparison of O–H, C=O, P–O, and Al–O bands across all three systems.

2.2.1.2. 1000–1350 cm^{-1} (P-region Normalization for OPGS and MOSG)

The region of 1000–1350 cm^{-1} was selected for normalization of the OPGS and MOSG spectra within the phosphate fingerprint region, based on the following rationale:

1) This window covers nearly all major phosphate-related vibrations of TXP-4:
   - P=O stretching (≈1250 cm^{-1} for OPGS; ≈1234 cm^{-1} for MOSG)
   - P–O–C and C–O–C stretching (≈ 1118 cm^{-1} for OPGS, partially overlapping with the ≈996 cm^{-1} coordination-related band in MOSG)

2) In this region, OPGS and MOSG exhibit strong and structurally informative absorption, while the aqueous gelling solution shows absorption close to the noise level. Thus, it functions effectively as a "phosphate-dominated fingerprint region" for comparing the evolution of the headgroup environment from OPGS to MOSG.

3) Compared with single-peak normalization (such as using only the P=O peak height), area-based normalization over 1000–1350 cm^{-1} takes into account the combined contributions of P=O and P–O–C / P–O–Al vibrations, providing a more comprehensive basis for analyzing intensity redistribution induced by organic–inorganic coordination.

Accordingly, 1000–1350 cm^{-1} was selected as the P-region normalization window for OPGS and MOSG to enable direct comparison of P=O shifts and the emergence of P–O–Al coordination bands under a unified phosphate-area baseline.

2.2.1.3 460–978 cm^{-1} (Al-region Normalization for AGS and MOSG)

The region of 460–978 cm^{-1} was chosen for normalization of AGS and MOSG within the aluminum–oxygen network region. The selection is justified as follows:

1) AGS exhibits a broad continuous band within 460–978 cm^{-1}, comprising:
   - 450–626 cm^{-1}: Al–O lattice vibrations and Al–O–Al bridging modes
   - 626–978 cm^{-1}: Al–OH bending vibrations and Al–O–H / Al–O–Al–OH related modes
2) In MOSG, this region shows pronounced peak redistribution and the appearance of new absorptions, such as:
   - ≈996 cm^{-1}: P–O–Al / Al–O–P coordination vibration
   - ≈624 cm^{-1}: reorganized aluminum–oxygen network vibration

This region therefore represents the primary response zone for the Al–O framework and Al–O–P coordination network.

3) OPGS exhibits extremely weak absorption (after diesel background subtraction) in this region and is therefore unsuitable for normalization.

Given these features, 460–978 cm^{-1} was selected as the Al-region normalization window for comparing the inorganic network evolution from AGS to MOSG, enabling clear observation of the spectral redistribution from broad Al–O / Al–OH features to coordinated Al–O–P structures.

2.2.2. Normalization Calculations

To allow direct comparison of spectral features across the different systems, area-based linear normalization was applied. Normalization affects only the absorbance

axis; the wavenumber axis and peak shapes are preserved.

2.2.2.1 Non-negative Clipping

Because baseline correction and background subtraction may introduce small negative absorbance values in non-absorbing regions, each spectrum was first subjected to non-negative clipping:

$$A_i^{+}(\tilde{\nu}) = \max\left(A_i(\tilde{\nu}),\ 0\right) \tag{S6}$$

where i represents OPGS, AGS, or MOSG.

All subsequent integrations and normalization factors were calculated using $A_i^{+}(\tilde{\nu})$.

2.2.2.2 Global Normalization for Three-phase Comparison (1400–1800 cm^{-1})

For each spectrum i, the integrated area $S_i^{(global)}$ within 1400–1800 cm^{-1} was computed as:

$$S_i^{(global)} = \int_{1400}^{1800} A_i^{+}(\tilde{\nu})\, d\tilde{\nu} \tag{S7}$$

The globally normalized spectrum $A_i^{(global)}(\tilde{\nu})$ is defined as:

$$A_i^{(global)}(\tilde{\nu}) = \frac{A_i^{+}(\tilde{\nu})}{S_i^{(global)}} \tag{S8}$$

After this process, all spectra have an integrated area of 1 within the 1400–1800 cm^{-1}global window, enabling direct comparison of O–H, C=O, P–O, and Al–O absorption intensities across 4000–400 cm^{-1}.

2.2.2.3. P-region Normalization for OPGS and MOSG (1000–1350 cm^{-1})

To focus on the evolution of the phosphate headgroup from OPGS to MOSG, the local area normalization within 1000–1350 cm^{-1} was applied.

$$S_i^{(\mathrm{P})} = \int_{1000}^{1350} A_i^{+}(\tilde{\nu})\, d\tilde{\nu}, i = OPGS\ or\ MOSG \tag{S9}$$

$$A_i^{(\mathrm{P-norm})}(\tilde{\nu}) = \frac{A_i^{+}(\tilde{\nu})}{S_i^{(\mathrm{P})}}\ , i = OPGS\ or\ MOSG \tag{S10}$$

The spectrum of AGS was excluded as its absorption in this region is negligible.

Comparing $A_{\mathrm{OPSG}}^{(\mathrm{P-norm})}$ and $A_{\mathrm{MOSG}}^{(\mathrm{P-norm})}$ enables analysis of P=O shifts and the emergence of P–O–Al coordination bands on a unified phosphate baseline.

2.2.2.4. Al-region Normalization for AGS and MOSG (460–978 cm^{-1})

To evaluate the reorganization of the inorganic aluminum–oxygen network, area normalization within 460–978 cm^{-1} was applied to AGS and MOSG:

$$S_i^{(\mathrm{Al})} = \int_{460}^{978} A_i^{+}(\tilde{\nu})\, d\tilde{\nu}, i = AGS\ or\ MOSG \tag{S11}$$

$$A_i^{(\mathrm{Al-norm})}(\tilde{\nu}) = \frac{A_i^{\dagger}(\tilde{\nu})}{S_i^{(\mathrm{Al})}}, i = AGS\ or\ MOSG \tag{S12}$$

The OPSG spectrum was excluded due to negligible signal in this region.

Comparison of $A_{\mathrm{AGS}}^{(\mathrm{Al-norm})}$ and $A_{\mathrm{MOSG}}^{(\mathrm{Al-norm})}$ allows quantitative analysis of band redistribution from broad Al–O / Al–OH features to coordinated Al–O–P vibrations at ~996 and ~624 cm^{-1}, supporting the spectral evidence for Al–O–P network formation in MOSG. Table S3 shows the integrated absorbance areas used for global, P-region, and Al-region normalization of the OPGS, AGS, and MOSG spectra.

Table S3. Integrated absorbance areas used for global, P-region, and Al-region normalization of the OPGS, AGS, and MOSG spectra.

Normalization type	Selected window(cm^{-1})	Peak area		
		OPGS	AGS	MOSG
Global normalization	1400–1800	7.95	7.34	10.04
P-region normalization	1000–1350	17.26	-	16.63
Al-region normalization	460–978	-	4.32	8.69
